# *doubletrouble*: an R/Bioconductor package for the identification, classification, and analysis of gene and genome duplications

**DOI:** 10.1093/bioinformatics/btaf043

**Published:** 2025-01-25

**Authors:** Fabricio Almeida-Silva, Yves Van de Peer

**Affiliations:** Department of Plant Biotechnology and Bioinformatics, Ghent University, Ghent 9052, Belgium; VIB Center for Plant Systems Biology, VIB, Ghent 9052, Belgium; Department of Plant Biotechnology and Bioinformatics, Ghent University, Ghent 9052, Belgium; VIB Center for Plant Systems Biology, VIB, Ghent 9052, Belgium; Department of Biochemistry, Genetics and Microbiology, Centre for Microbial Ecology and Genomics, University of Pretoria, Pretoria 0028, South Africa; College of Horticulture, Academy for Advanced Interdisciplinary Studies, Nanjing Agricultural University, Nanjing 210095, Nanjing, China

## Abstract

**Summary:**

Gene and genome duplications are major evolutionary forces that shape the diversity and complexity of life. However, different duplication modes have distinct impacts on gene function, expression, and regulation. Existing tools for identifying and classifying duplicated genes are either outdated or not user-friendly. Here, we present *doubletrouble*, an R/Bioconductor package that provides a comprehensive and robust framework for analyzing duplicated genes from genomic data. *doubletrouble* can detect and classify gene pairs as derived from six duplication modes (segmental, tandem, proximal, retrotransposon-derived, DNA transposon-derived, and dispersed duplications), calculate substitution rates, detect signatures of putative whole-genome duplication events, and visualize results as publication-ready figures. We applied *doubletrouble* to classify the duplicated gene repertoire in 822 eukaryotic genomes, and results were made available through a user-friendly web interface.

**Availability and implementation:**

*doubletrouble* is available on Bioconductor (https://bioconductor.org/packages/doubletrouble), and the source code is available in a GitHub repository (https://github.com/almeidasilvaf/doubletrouble). doubletroubledb is available online at https://almeidasilvaf.github.io/doubletroubledb/.

## 1 Introduction

Gene and genome duplications are an important source of novel genetic material for evolution to work with (Ohno 1970). However, gene and genome duplications contribute to genome evolution in different ways, and genes created by these two mechanisms evolve under different selection pressures, and display different evolutionary trajectories at the genomic, transcriptomic, and epigenomic levels (Casneuf *et al.* 2006, Freeling and Thomas 2006, Ganko *et al.* 2007, Hakes *et al.* 2007, Freeling 2009, De Smet *et al.* 2013, Li *et al.* 2016, Qiao *et al.* 2018, 2019, Almeida-Silva *et al.* 2020, Almeida-Silva and Van de Peer 2023a,b, Kenchanmane Raju *et al.* 2023, Lallemand *et al.* 2023). Furthermore, depending on the duplication mode, gene retention can be biased, with some gene classes (e.g. transcription factors and members of protein complexes) being preferentially retained after whole-genome duplication (Freeling and Thomas 2006, Birchler and Veitia 2010).

To understand the differential contribution of each duplication mode to genome evolution and the emergence of novel traits, researchers typically use a utility program in the MCScanX toolkit (Wang *et al.* 2012) to classify gene pairs as derived from segmental, tandem, proximal, or dispersed duplications. However, MCScanX is no longer actively maintained, and its classification scheme does not include transposon-derived duplications. The *DupGen_finder* pipeline (Qiao *et al.* 2019) extends the classification scheme in MCScanX to identify transposon-derived duplicates, but it consists of a set of Perl scripts, not a distributable software package (i.e. available from a package manager, with unit tests, continuous integration, and documentation), hindering its stability and usability.

Here, we introduce *doubletrouble*, an R/Bioconductor package to identify, classify, and analyze duplicated genes from genomic data. *doubletrouble* can identify and classify duplicated gene pairs as derived from segmental, tandem, proximal, retrotransposon-derived, DNA transposon-derived, and dispersed duplications. Duplicate pairs identified and classified with *doubletrouble* can be further used to calculate substitution rates (K_a_, K_s_, and their ratio K_a_/K_s_), investigate signatures of potential whole-genome duplication events, and generate publication-ready figures. We demonstrate *doubletrouble’*s effectiveness by identifying and classifying the entire duplicated gene repertoire in 822 eukaryotic genomes from Ensembl instances. Finally, to facilitate data reuse, we created a web application (https://almeidasilvaf.github.io/doubletroubledb) where users can explore and download data generated here on the duplicated gene repertoire across the Eukarya tree of life.

## 2 Implementation

*doubletrouble* is part of the Bioconductor ecosystem of R packages and, as such, was designed to easily interoperate with other Bioconductor packages. For that, data classes used in *doubletrouble* are either base R classes or core Bioconductor S4 classes, such as *GRanges* objects to store genomic annotation (Lawrence *et al.* 2013), and *AA/DNAStringSet* objects to store sequence data (Pagès *et al.* 2024).

### 2.1 Data input and processing

The required input data are whole-genome protein sequences (one per gene, primary transcript only) as *AAStringSet* objects, and gene annotation as *GRanges* objects, both in lists with matching names representing species names or any other genome identifiers. Users can create such lists of *AAStringSet* and *GRanges* objects from multiple FASTA and GFF/GTF files in a directory with the functions *fasta2AAStringSetlist()* and *gff2GRangesList()*, available in the R package syntenet (Almeida-Silva *et al.* 2023). Sequence and annotation data must be processed with the function *process_input()* from the syntenet package to ensure that only one sequence per gene is present (see Almeida-Silva *et al.* 2023 for details).

### 2.2 Identification and classification of duplicated gene pairs

Processed data can be used as input to the function *run_diamond()* from the syntenet package to perform intraspecies similarity searches with DIAMOND (Buchfink *et al.* 2021) (default parameters: top hits = 5; E-value = 1e−10). Alternatively, users who wish to use a different similarity search program [e.g. BLAST (Altschul *et al.* 1997) or MMseqs2 (Steinegger and Söding 2017)] can export processed sequences to FASTA files with the function *export_sequences()*, run their favorite program on the command line, and read its tabular output with the function *read_diamond()*. The tabular output of DIAMOND and similar programs is considered to contain all paralogous gene pairs within a genome (i.e. a “paranome”) (Emms and Kelly 2019, Qiao *et al.* 2019, Sensalari *et al.* 2022), but users can also perform further filtering (e.g. by coverage, similarity, etc.) to refine results if they want to be more stringent.

**Figure 1.**
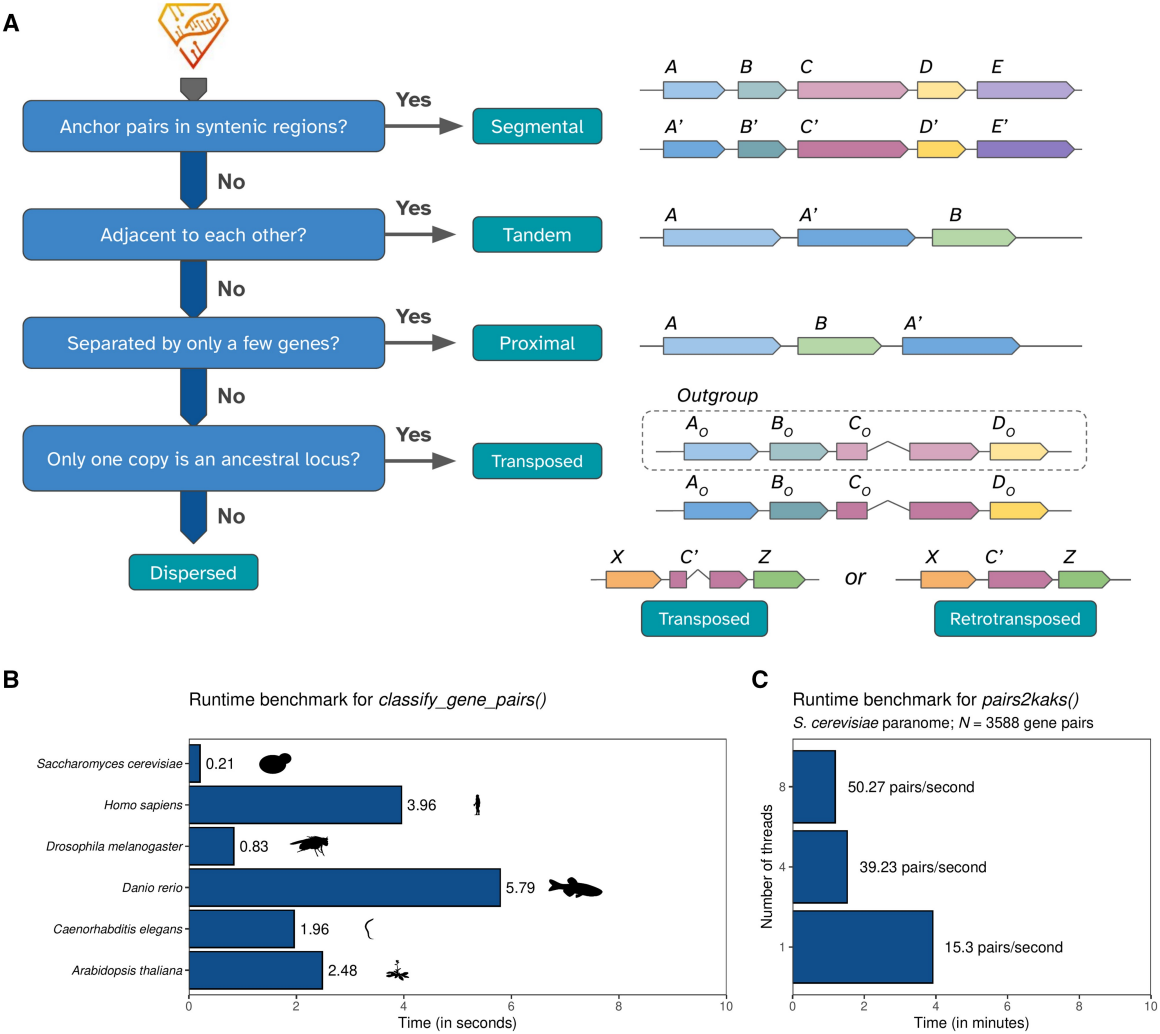
Schematic overview of the classification algorithm for duplicated genes, and runtime benchmarks. (A) Algorithm for the classification of duplicates. From the output of DIAMOND or related sequence similarity search tools, duplicated gene pairs are classified according to duplication mode in a stepwise manner. Gene pairs that fail to meet the criteria for all duplication modes will be classified as “dispersed duplicates,” which is not a true duplication mechanism, denoting that their duplication mechanism is actually unknown. (B) Runtime benchmark of the function classify_gene_pairs() for model organisms using the “standard” scheme. Silhouettes were obtained from PhyloPic (https://www.phylopic.org/) using the R package rphylopic (Gearty and Jones 2023). (C) Runtime benchmark of the function pairs2kaks() with one, four, and eight threads. For each gene pair, this function performs a pairwise alignment of the sequences, and then calculates substitution rates.

The paranomes and gene annotations for each species are used as input to the function *classify_gene_pairs()*, which classifies paralogs by mode of duplication based on four possible classification schemes (Fig. 1A). In the binary scheme, paralogs are classified as derived from either segmental duplications (SD) or small-scale duplications (SSD). The standard scheme (default) further classifies SSD-derived pairs as originating from tandem (TD), proximal (PD), or dispersed duplications (DD). The extended scheme includes transposon-derived duplications (TRD) as an additional duplication mode, and the full scheme further classifies TRD-derived genes as originating from either retrotransposons (rTRD) or DNA transposons (dTRD).

To classify paralogs by duplication mode, the function *classify_gene_pairs()* uses the syntenet package to detect intragenomic syntenic regions. Paralogs that are anchor pairs in syntenic regions are classified as segmental duplicates (SD), and all other duplicates are classified as small-scale duplicates (SSD). SSD pairs that are physically adjacent to each other in the genome are classified as tandem duplicates (TD), SSD pairs that are separated by only a few genes (default = 10, but adjustable) are classified as proximal duplicates (PD), and all other duplicates are classified as dispersed duplicates (DD). To further classify DD pairs as originating from transposon-derived duplications (TRD), users must provide a table with similarity search results between all query species and an outgroup [e.g. as created with the *run_diamond()* function], which will be used to detect interspecies syntenic regions. DD pairs for which only one copy is an ancestral locus (i.e. syntenic with the outgroup species) are classified as transposed duplicates (TRD). If multiple species are passed as an outgroup, pairs that are transposed relative to a minimum percentage of outgroup species (70% by default, but adjustable) are classified as TRD. Finally, TRD pairs for which only one copy is intronless are classified as retrotransposed duplicates (rTRD), while other TRD pairs are classified as DNA transposon-derived duplicates (dTRD).

As genes can be duplicated multiple times, the same gene can appear in multiple duplicate pairs in the output of *classify_gene_pairs()*, often derived from different duplication modes. However, users sometimes want to assign unique modes of duplication to individual genes (e.g. to understand the origins of particular genes), as implemented in *DupGen_finder* (Qiao *et al.* 2018, 2019, Moharana and Venancio 2020). This can be performed with the function *classify_genes()*, which uses classified gene pairs returned by *classify_gene_pairs()* to assign genes to unique duplication modes using the following hierarchy: SD > TD > PD > rTRD > dTRD > DD.

### 2.3 Calculation of substitution rates per substitution site

Rates of synonymous substitution per substitution site (*K*_s_), nonsynonymous substitutions per substitution site (*K*_a_), and their ratios (*K*_a_/*K*_s_) for each gene pair can be calculated with the function *pairs2kaks()* using all 17 codon-based models of nucleotide substitution from KaKs_Calculator 2.0 (Wang *et al.* 2010), as implemented in MSA2dist (Ullrich 2024). Peaks in Ks distributions, which typically suggest the presence of whole-genome duplication (WGD) events, can be predicted with normal and lognormal mixture modeling using the function *find_ks_peaks()*. However, we advise users to interpret such peaks with caution, as mixture models are prone to overclustering and overfitting (Tiley *et al.* 2018). Mixture components can further be used to classify gene pairs in age groups with the function *split_pairs_by_peak()*, with age boundaries defined by the intersection point between two different components or by the mean ± *N* standard deviations (default = 2, but can be adjusted). Parameters of mixture components (e.g. mean and standard deviation) and age groups can also be used to distinguish which segmental duplicates (SD) likely originated from WGD events, and which likely originated from duplications of large genomic regions (e.g. entire chromosomes or chromosomal segments).

### 2.4 Data visualization

Graphical functions are available to create publication-ready plots from the output of *doubletrouble* functions. Absolute and relative frequencies of duplicated genes/gene pairs by mode per species can be visualized with the function *plot_duplicate_freqs()*. Distributions of substitution rates (*K*_a_, *K*_s_, and *K*_a_/*K*_s_) per species can be visualized with the function *plot_rates_by_species()*. Likewise, *K*s distributions and peaks can be visualized with the function *plot_ks_distro()* and *plot_ks_peaks()*, respectively. All plots are created with the ggplot2 system (Wickham 2016), allowing users to customize plot esthetics (e.g. color palettes, axis labels, font aspects, themes, etc.).

### 2.5 Benchmark datasets

Whole-genome protein sequences and gene annotations were obtained for all species in release 57 of Ensembl Fungi (*N *=* *70), Ensembl Protists (*N *=* *33), Ensembl Plants (*N *=* *149) and Ensembl Metazoa (*N *=* *253) (Yates *et al.* 2022), and in release 110 of Ensembl (*N *=* *317) (Martin *et al.* 2023), leading to a total of 822 genomes across the Eukarya tree of life (Supplementary Table S1). BUSCO scores (Manni *et al.* 2021) for each species were obtained and visualized using the Bioconductor package cogeqc (Almeida‐Silva and Van de Peer 2023a,b). BUSCO genes shared by >90% of the species were aligned with MAFFT (Katoh and Standley 2013), and multiple sequence alignments were concatenated and trimmed to remove alignment columns with >50% gaps. Filtered supermatrices were used for phylogeny inference with IQ-TREE2 (Minh *et al.* 2020). Oomycetes, red algae, *Giardia lamblia*, *Mnemiopsis leidyi*, and *Saccharomyces cerevisiae* were used as outgroups for Ensembl Fungi, Ensembl Plants, Ensembl Protists, Ensembl Metazoa, and Ensembl, respectively (Supplementary Text S1).

## 3 Results and discussion

### 3.1 Runtime benchmark and comparison to existing tools

To assess *doubletrouble’*s performance, we classified duplicate pairs with *classify_gene_pairs()* in the genomes of six model organisms, namely budding yeast (*Saccharomyces cerevisiae*), humans (*Homo sapiens*), fruit fly (*Drosophila melanogaster*), zebrafish (*Danio rerio*), worm (*Caenorhabditis elegans*), and thale cress (*Arabidopsis thaliana*) (Supplementary Text S5). On an Ubuntu 22.04 laptop with an Intel i5-1345U processor (4.7 GHz; 16 GB RAM), duplicate classification took from 0.21 s (*S. cerevisiae*) to 5.79 s (*D. rerio*), revealing that such task is efficient and can be easily performed on large-scale genomic datasets (Fig. 1B). Next, we calculated substitution rates for all duplicated gene pairs in the *S. cerevisiae* genome (*N *=* *3588). Using a single thread, the function *pairs2kaks()* took 3.9 minutes to complete (∼15 pairs/s; Fig. 1C), which includes the time to perform a pairwise alignment and calculate substitution rates for each gene pair. When parallelization was enabled with four and eight threads, calculations ran in 1.52 minutes (∼39 pairs/s) and 1.19 minutes (∼50 pairs/s), respectively (Fig. 1C).

Next, we compared *doubletrouble* to the *DupGen_finder* pipeline (Qiao *et al.* 2019) to assess if and how they differ in terms of runtime and classification results. For that, we classified duplicate pairs in the *Arabidopsis thaliana* genome using *Amborella trichopoda* as outgroup (Supplementary Text S5), and we used the “extended” classification scheme of *doubletrouble*, which is equivalent to *DupGen_finder*. We observed no significant difference in runtime, with both implementations taking around 3 s to classify duplicates. The frequencies of duplicate pairs per mode were similar for SD, TD, and TRD pairs, but we found profound differences in the numbers of PD and DD pairs (N_PD_ = 2789 and 896, and N_DD_ = 31 589 and 17 834 for *doubletrouble* and *DupGen_finder*, respectively). Strikingly, of the 47 485 paralogous pairs identified by DIAMOND searches (after filtering by E-value, and removing self and redundant hits), *DupGen_finder* returned only 31 157 classified pairs, indicating that it probably performed some undocumented filtering before classification (Supplementary Text S5). Besides, since *doubletrouble* detected many more PD pairs than *DupGen_finder*, we checked whether such a difference was due to misclassification by *doubletrouble*. We observed that PD pairs detected exclusively by *doubletrouble* were physically close to each other, separated by only a few genes (<10), indicating that they are true PD pairs that *DupGen_finder* either failed to classify or filtered out (Supplementary Text S5).

### 3.2 Use cases: gene duplications in the eukaryotic domain, and polyploidization in legumes

To demonstrate the effectiveness of *doubletrouble*, we obtained genomic data for 822 species in Ensembl and Ensembl Genomes (Fungi, Protists, Plants, and Metazoa), identified duplicated gene pairs, and classified them by duplication mode using the “full” classification scheme (Supplementary Text S2). Expectedly, we observed a much larger proportion of SD-derived genes in plants as compared to all other taxa (Supplementary Text S4), which is likely due to pervasive WGD events in the plant tree of life (Van de Peer *et al.* 2017, Ren *et al.* 2018, One Thousand Plant Transcriptomes Initiative 2019). Using a dataset of legume genomes, we also demonstrated the importance of using only segmental duplicates to infer WGD events, as whole-paranome K_s_ distributions can lead to inaccurate WGD predictions (Supplementary Texts S3 and S4).

### 3.3 A web application toward FAIR data principles

To ensure the data generated in this study are FAIR (Findable, Accessible, Interoperable, and Reusable), we developed *doubletroubledb*, a user-friendly web application where users can explore frequencies of duplicated genes for all 822 eukaryote species (see Materials and Methods for details). The web application also works as an interface to download tabular data files with duplicated genes and gene pairs for each species. The application is accessible at https://almeidasilvaf.github.io/doubletroubledb, and the source code is accessible at https://github.com/almeidasilvaf/doubletroubledb.

### 3.4 Known limitation

*doubletrouble* classifies duplicate pairs by duplication mode using paranomes obtained with DIAMOND (or other sequence similarity search programs). However, identifying paralogs based on sequence similarity can lead to false positives in organisms with frequent horizontal gene transfers (e.g. prokaryotes). Thus, for such cases, we recommend using tools that can distinguish paralogs from xenologs (i.e. homologs derived from horizontal gene transfer), and then using paralogs-only for classification with *doubletrouble*.

## 4 Conclusion

*doubletrouble* is an R/Bioconductor package that can be used to identify and classify duplicated genes by duplication mode, calculate substitution rates for gene pairs, and create publication-ready figures. This package should help in exploring the duplication landscape in different species, and the datasets generated in this study will be an important resource for researchers studying the evolution of gene duplication in eukaryotes.

## Supplementary Material

btaf043_Supplementary_Data

## Data Availability

All data and code used in this article are available in its online Supplementary Material and at https://github.com/almeidasilvaf/doubletrouble_paper.
