# Supplementary material for: doubletrouble: an R/Bioconductor package for the identification, classification, and analysis of gene and genome duplications
Source: Bioinformatics. 2025 Jan 25;41(2):btaf043. doi: 10.1093/bioinformatics/btaf043 (PMC11810640; doi:10.1093/bioinformatics/btaf043)
Supplement: btaf043_Supplementary_Data [file btaf043_supplementary_data.zip › ad438_supplementary_texts_combined.pdf]

Supplementary Text S1: Obtaining species trees for Ensembl instances

Fabricio Almeida-Silva[1] **and Yves Van de Peer**[1]

[1]VIB-UGent Center for Plant Systems Biology, Ghent University, Ghent, Belgium

27 February 2024

Contents

1 Introduction 2
2 Summary stats 4
3 Getting species metadata 4
4 BUSCO-guided phylogeny inference 7
4.1 Obtaining BUSCO sequences 7
4.2 Tree inference from BUSCO genes 13
5 Obtaining BUSCO scores 16
Session info 17

1 Introduction

Here, we will describe the code to obtain a species tree for each Ensembl instance using BUSCO genes.

```
library(here)
## here() starts at /home/faalm/Dropbox/package_benchmarks/doubletrouble_paper
library(tidyverse)
## -- Attaching core tidyverse packages ------------------------ tidyverse 2.0.0 --
## v dplyr     1.1.4     v readr     2.1.5
## v forcats   1.0.0     v stringr   1.5.1
## v ggplot2   3.4.4     v tibble    3.2.1
## v lubridate 1.9.3     v tidyr     1.3.1
## v purrr     1.0.2
## -- Conflicts ------------------------------------------ tidyverse_conflicts() --
## x dplyr::filter() masks stats::filter()
## x dplyr::lag()    masks stats::lag()
## i Use the conflicted package (<http://conflicted.r-lib.org/>) to force all conflicts to become errors
library(biomaRt)
library(Herper)
## Loading required package: reticulate
##
## Attaching package: 'Herper'
##
## The following object is masked from 'package:reticulate':
##
##     conda_search
library(taxize)
library(Biostrings)
## Loading required package: BiocGenerics
##
## Attaching package: 'BiocGenerics'
##
## The following objects are masked from 'package:lubridate':
##
##     intersect, setdiff, union
##
## The following objects are masked from 'package:dplyr':
##
##     combine, intersect, setdiff, union
##
## The following objects are masked from 'package:stats':
##
##     IQR, mad, sd, var, xtabs
##
## The following objects are masked from 'package:base':
##
##     anyDuplicated, aperm, append, as.data.frame, basename, cbind,
##     colnames, dirname, do.call, duplicated, eval, evalq, Filter, Find,
##     get, grep, grepl, intersect, is.unsorted, lapply, Map, mapply,
##     match, mget, order, paste, pmax, pmax.int, pmin, pmin.int,
```

```
##     Position, rank, rbind, Reduce, rownames, sapply, setdiff, sort,
##     table, tapply, union, unique, unsplit, which.max, which.min
##
## Loading required package: S4Vectors
## Loading required package: stats4
##
## Attaching package: 'S4Vectors'
##
## The following objects are masked from 'package:lubridate':
##
##     second, second<-
##
## The following objects are masked from 'package:dplyr':
##
##     first, rename
##
## The following object is masked from 'package:tidyr':
##
##     expand
##
## The following object is masked from 'package:utils':
##
##     findMatches
##
## The following objects are masked from 'package:base':
##
##     expand.grid, I, unname
##
## Loading required package: IRanges
##
## Attaching package: 'IRanges'
##
## The following object is masked from 'package:lubridate':
##
##     %within%
##
## The following objects are masked from 'package:dplyr':
##
##     collapse, desc, slice
##
## The following object is masked from 'package:purrr':
##
##     reduce
##
## Loading required package: XVector
##
## Attaching package: 'XVector'
##
## The following object is masked from 'package:purrr':
##
##     compact
```

```
##
## Loading required package: GenomeInfoDb
##
## Attaching package: 'Biostrings'
##
## The following object is masked from 'package:base':
##
##     strsplit
library(cogeqc)

set.seed(123) # for reproducibility
options(timeout = 1e6) # to allow download of big files

source(here("code", "utils.R"))
source(here("code", "utils_busco_phylogeny.R"))
```

2 Summary stats

To start with, let's get the number of species for each instance:

```
# Get number of species in Ensembl Genomes
instances <- c("fungi_mart", "plants_mart", "metazoa_mart", "protists_mart")
nspecies_ensemblgenomes <- unlist(lapply(instances, function(x) {
    return(nrow(listDatasets(useEnsemblGenomes(biomart = x))))
}))

# Get number of species in Ensembl
nspecies_ensembl <- nrow(listDatasets(useEnsembl(biomart = "genes")))

# Combine summary stats onto a data frame
nspecies_all <- data.frame(
    instance = c(gsub("_mart", "", instances), "ensembl"),
    n_genes = c(nspecies_ensemblgenomes, nspecies_ensembl)
)

nspecies_all
##   instance n_genes
## 1    fungi      70
## 2   plants     151
## 3  metazoa     280
## 4 protists      33
## 5  ensembl     214
```

3 Getting species metadata

Now, let's get species metadata for each Ensembl instance.

```r
# Metadata column names
col_names <- c(
    "name", "species", "division", "taxonomy_id", "assembly",
    "assembly_accession", "genebuild", "variation", "microarray", "pan_compara",
    "peptide_compara", "genome_alignments", "other_alignments", "core_db",
    "species_id"
)

to_remove <- c(
    "variation", "microarray", "pan_compara", "peptide_compara",
    "genome_alignments", "other_alignments", "core_db", "species_id"
)

# Ensembl Fungi
metadata_fungi <- read_tsv(
    "http://ftp.ebi.ac.uk/ensemblgenomes/pub/release-57/fungi/species_EnsemblFungi.txt",
    col_names = col_names, skip = 1, col_select = 1:15, show_col_types = FALSE
) |>
    dplyr::filter(!startsWith(core_db, "fungi_")) |>
    dplyr::select(!any_of(to_remove)) |>
    as.data.frame()

metadata_fungi <- cbind(
    metadata_fungi,
    classification(metadata_fungi$taxonomy_id, db = "ncbi") |>
        format_classification()
)

# Ensembl Plants
metadata_plants <- read_tsv(
    "http://ftp.ebi.ac.uk/ensemblgenomes/pub/release-57/plants/species_EnsemblPlants.txt",
    col_names = col_names, skip = 1, col_select = 1:15, show_col_types = FALSE
) |>
    dplyr::filter(species != "triticum_aestivum_kariega") |>
    dplyr::select(!any_of(to_remove)) |>
    as.data.frame()

metadata_plants <- cbind(
    metadata_plants,
    classification(metadata_plants$taxonomy_id, db = "ncbi") |>
        format_classification()
)

# Ensembl Metazoa
metadata_metazoa <- read_tsv(
    "http://ftp.ebi.ac.uk/ensemblgenomes/pub/release-57/metazoa/species_EnsemblMetazoa.txt",
    col_names = col_names, skip = 1, col_select = 1:15, show_col_types = FALSE
) |>
    dplyr::filter(!startsWith(core_db, "metazoa_")) |>
    dplyr::select(!any_of(to_remove)) |>
    as.data.frame()
```

```
metadata_metazoa <- cbind(
    metadata_metazoa,
    classification(metadata_metazoa$taxonomy_id, db = "ncbi") |>
        format_classification()
)

# Ensembl Protists
metadata_protists <- read_tsv(
    "http://ftp.ebi.ac.uk/ensemblgenomes/pub/release-57/protists/species_EnsemblProtists.txt",
    col_names = col_names, skip = 1, col_select = 1:15, show_col_types = FALSE
) |>
    dplyr::filter(!startsWith(core_db, "protists_")) |>
    dplyr::select(!any_of(to_remove)) |>
    as.data.frame()

metadata_protists <- cbind(
    metadata_protists,
    classification(metadata_protists$taxonomy_id, db = "ncbi") |>
        format_classification()
)

# Ensembl
metadata_ensembl <- read_tsv(
    "https://ftp.ensembl.org/pub/release-110/species_EnsemblVertebrates.txt",
    col_names = col_names, skip = 1, col_select = 1:15, show_col_types = FALSE
) |>
    dplyr::select(!any_of(to_remove)) |>
    as.data.frame()

metadata_ensembl <- cbind(
    metadata_ensembl,
    classification(metadata_ensembl$taxonomy_id, db = "ncbi") |>
        format_classification()
)

# Combining all metadata data frames into a list and saving it
metadata_all <- list(
    fungi = metadata_fungi,
    plants = metadata_plants,
    metazoa = metadata_metazoa,
    protists = metadata_protists,
    ensembl = metadata_ensembl
)

save(
    metadata_all, compress = "xz",
    file = here("products", "result_files", "metadata_all.rda")
)
```

4 BUSCO-guided phylogeny inference

Here, for each Ensembl instance, we infer a species tree using the following workflow:

1. Run BUSCO in protein mode with **cogeqc**, using translated sequences for primary transcripts as input;
2. Get the sequences of the identified complete BUSCOs that are shared across all species;
3. Perform a multiple sequence alignment for each BUSCO gene family.
4. Trim the alignments to remove columns with >50% of gaps.
5. Infer a phylogeny with IQ-TREE2.

To start with, we will use the Bioconductor package **Herper** to create a Conda environment containing BUSCO and all its dependencies. Then, we will use this environment to run BUSCO from the R session.

```
# Create Conda environment with BUSCO
my_miniconda <- "~/"

conda <- install_CondaTools(
    tools = "busco==5.5.0",
    env = "busco_env",
    pathToMiniConda = my_miniconda
)
```

4.1 Obtaining BUSCO sequences

To obtain sequences for BUSCO genes, we will run BUSCO in protein mode using the R/Bioconductor package **cogeqc**. Then, we will read the sequences for complete, single-copy BUSCOs, and keep only BUSCO genes that are shared by a certain % of the species. Ideally, this cut-off should be 100% of conservation (i.e., the BUSCO gene is found in all species), but it can be relaxed for some clades.

4.1.1 Ensembl Fungi

Here, we will obtain BUSCO genes for Ensembl Fungi species using the following parameters:

1. Lineage: **eukaryota_odb10**
2. Conservation: 100%

```
# Download whole-genome protein sequences to a directory sequences
busco_fungi <- file.path("~/Downloads/busco_fungi")
seq_fungi <- file.path(busco_fungi, "seqs")
if(!dir.exists(seq_fungi)) { dir.create(seq_fungi, recursive = TRUE) }

download_filtered_proteomes(metadata_all$fungi, "fungi", seq_fungi)

# Run BUSCO in `protein` mode
with_CondaEnv(
    "busco_env",
    cogeqc::run_busco(
        sequence = seq_fungi,
        outlabel = "ensembl_fungi",
```

```
        mode = "protein",
        lineage = "eukaryota_odb10",
        outpath = busco_fungi,
        threads = 3,
        download_path = busco_fungi

    ),
    pathToMiniConda = my_miniconda
)

outdir <- file.path(busco_fungi, "ensembl_fungi")
fungi_busco_seqs <- read_busco_sequences(outdir, verbose = TRUE)
```

Saving BUSCO sequences:

```
# Save list of AAStringSet objects with conserved BUSCO sequences
save(
    fungi_busco_seqs, compress = "xz",
    file = here("products", "result_files", "busco_seqs", "fungi_busco_seqs.rda")
)
```

4.1.2 Ensembl Plants

Here, we will use the lineage data set **eukaryota_odb10**. We could use **viridiplantae_odb10**, but there are 3 Rhodophyta species (*Chondrus crispus*, *Galdieria sulphuraria*, and *Cyanidioschyzon merolae*). Because none of the BUSCO genes were shared by all species, we selected genes shared by >60% of the species, and then manually selected BUSCO genes in a way that all species are included. This was required because some taxa (in particular *Triticum* species) had very few BUSCO genes.

```
# Download whole-genome protein sequences to a directory sequences
busco_plants <- file.path("~/Downloads/busco_plants")
seq_plants <- file.path(busco_plants, "seqs")
if(!dir.exists(seq_plants)) { dir.create(seq_plants, recursive = TRUE) }

download_filtered_proteomes(metadata_all$plants, "plants", seq_plants)

# Run BUSCO in `protein` mode
with_CondaEnv(
    "busco_env",
    cogeqc::run_busco(
        sequence = seq_plants,
        outlabel = "ensemblplants",
        mode = "protein",
        lineage = "eukaryota_odb10",
        outpath = busco_plants,
        threads = 4,
        download_path = busco_plants

    ),
    pathToMiniConda = my_miniconda
```

```
)

# Read sequences of BUSCOs preserved in >=60% of the species
outdir <- file.path(busco_plants, "ensemblplants")
plants_busco_seqs <- read_busco_sequences(outdir, conservation_freq = 0.6)

# Select 10 BUSCO genes so that all species are represented
plants_busco_pav <- get_busco_pav(plants_busco_seqs)

#' The following code was used to manually select BUSCOs in a way that
#' all species are represented

#> ht <- ComplexHeatmap::Heatmap(plants_busco_pav)
#> ht <- ComplexHeatmap::draw(ht)
#> InteractiveComplexHeatmap::htShiny(ht)


# Create a vector of selected BUSCOs
selected_buscos <- c(
    "549762at2759", "1003258at2759", "1247641at2759",
    "1200489at2759", "1398309at2759", "1346432at2759",
    "1266231at2759", "1094121at2759", "1421503at2759",
    "664730at2759", "1405073at2759", "450058at2759",
    "865202at2759", "901894at2759", "1450538at2759",
    "1284731at2759"
)

# Subset sequences to keep only selected BUSCOs
plants_busco_seqs <- plants_busco_seqs[selected_buscos]
```

Saving BUSCO sequences:

```
# Save list of AAStringSet objects with conserved BUSCO sequences
save(
    plants_busco_seqs, compress = "xz",
    file = here("products", "result_files", "busco_seqs", "plants_busco_seqs.rda")
)
```

4.1.3 Ensembl Protists

Here, we will obtain BUSCO genes for Ensembl Protists species using the following parameters:

1. Lineage: **eukaryota_odb10**
2. Conservation: 100%

```
# Download whole-genome protein sequences to a directory sequences
busco_protists <- file.path("~/Downloads/busco_protists")
seq_protists <- file.path(busco_protists, "seqs")
if(!dir.exists(seq_protists)) { dir.create(seq_protists, recursive = TRUE) }

download_filtered_proteomes(metadata_all$protists, "protists", seq_protists)
```

```r
# Run BUSCO in `protein` mode
with_CondaEnv(
    "busco_env",
    cogeqc::run_busco(
        sequence = seq_protists,
        outlabel = "ensemblprotists",
        mode = "protein",
        lineage = "eukaryota_odb10",
        outpath = busco_protists,
        threads = 4,
        download_path = busco_protists

    ),
    pathToMiniConda = my_miniconda
)

# Read sequences of BUSCOs preserved in >=60% of the species
outdir <- file.path(busco_protists, "ensemblprotists")
protists_busco_seqs <- read_busco_sequences(outdir, verbose = TRUE)
```

Saving BUSCO sequences:

```r
# Save list of AAStringSet objects with conserved BUSCO sequences
save(
    protists_busco_seqs, compress = "xz",
    file = here("products", "result_files", "busco_seqs", "protists_busco_seqs.rda")
)
```

4.1.4 Ensembl Metazoa

For the Metazoa instance, we used the *metazoa_odb10* lineage data set.

```r
# Download whole-genome protein sequences to a directory sequences
busco_metazoa <- file.path("~/Downloads/busco_metazoa")
seq_metazoa <- file.path(busco_metazoa, "seqs")
if(!dir.exists(seq_metazoa)) { dir.create(seq_metazoa, recursive = TRUE) }

download_filtered_proteomes(metadata_all$metazoa, "metazoa", seq_metazoa)

# Run BUSCO in `protein` mode
with_CondaEnv(
    "busco_env",
    cogeqc::run_busco(
        sequence = seq_metazoa,
        outlabel = "ensemblmetazoa",
        mode = "protein",
        lineage = "metazoa_odb10",
        outpath = busco_metazoa,
        threads = 4,
        download_path = busco_metazoa
```

```
    ),
    pathToMiniConda = my_miniconda
)

# Read sequences of BUSCOs preserved in >=60% of the species
outdir <- file.path(busco_metazoa, "ensemblmetazoa")
metazoa_busco_seqs <- read_busco_sequences(outdir, conservation_freq = 0.9)

# Select 10 BUSCO genes so that all species are represented
metazoa_busco_pav <- get_busco_pav(metazoa_busco_seqs)

#' The following code was used to manually select BUSCOs in a way that
#' all species are represented

#> ht <- ComplexHeatmap::Heatmap(metazoa_busco_pav)
#> ht <- ComplexHeatmap::draw(ht)
#> InteractiveComplexHeatmap::htShiny(ht)


# Create a vector of selected BUSCOs
selected_buscos <- c(
    "351226at33208", "135294at33208",
    "517525at33208", "501396at33208",
    "464987at33208", "443518at33208",
    "495100at33208", "335107at33208",
    "454911at33208", "134492at33208"
)

# Subset sequences to keep only selected BUSCOs
metazoa_busco_seqs <- metazoa_busco_seqs[selected_buscos]
```

Saving BUSCO sequences:

```
# Save list of AAStringSet objects with conserved BUSCO sequences
save(
    metazoa_busco_seqs, compress = "xz",
    file = here("products", "result_files", "busco_seqs", "metazoa_busco_seqs.rda")
)
```

4.1.5 Ensembl Vertebrates

Here, because there are 3 non-vertebrate species (*C. elegans*, *D. melanogaster*, and *S. cerevisiae*), we will use the lineage data set **eukaryota_odb10**.

```
# Download whole-genome protein sequences to a directory sequences
busco_vertebrates <- file.path("~/Downloads/busco_vertebrates")
seq_vertebrates <- file.path(busco_vertebrates, "seqs")
if(!dir.exists(seq_vertebrates)) { dir.create(seq_vertebrates, recursive = TRUE) }

download_filtered_proteomes(metadata_all$ensembl, "ensembl", seq_vertebrates)
```

```r
# Run BUSCO in `protein` mode
with_CondaEnv(
    "busco_env",
    cogeqc::run_busco(
        sequence = seq_vertebrates,
        outlabel = "ensemblvertebrates",
        mode = "protein",
        lineage = "eukaryota_odb10",
        outpath = busco_vertebrates,
        threads = 4,
        download_path = busco_vertebrates

    ),
    pathToMiniConda = my_miniconda
)

# Read sequences of BUSCOs preserved in >=90% of the species
outdir <- file.path(busco_vertebrates, "ensemblvertebrates")
vertebrates_busco_seqs <- read_busco_sequences(outdir, conservation_freq = 0.9)

# Select 10 BUSCO genes so that all species are represented
vertebrates_busco_pav <- get_busco_pav(vertebrates_busco_seqs)

#' The following code was used to manually select BUSCOs in a way that
#' all species are represented

#> ht <- ComplexHeatmap::Heatmap(vertebrates_busco_pav)
#> ht <- ComplexHeatmap::draw(ht)
#> InteractiveComplexHeatmap::htShiny(ht)


# Create a vector of selected BUSCOs
selected_buscos <- c(
    "834694at2759", "551907at2759",
    "491869at2759", "1085752at2759",
    "801857at2759", "1398309at2759",
    "176625at2759", "1324510at2759",
    "1377237at2759", "1085752at2759"
)

# Subset sequences to keep only selected BUSCOs
vertebrates_busco_seqs <- vertebrates_busco_seqs[selected_buscos]
```

Saving BUSCO sequences:

```r
# Save list of AAStringSet objects with conserved BUSCO sequences
save(
    vertebrates_busco_seqs, compress = "xz",
    file = here("products", "result_files", "busco_seqs", "vertebrates_busco_seqs")
)
```

4.2 Tree inference from BUSCO genes

Now, we will infer species trees from MSAs for each family, and from a single concatenated MSA (when possible).

4.2.1 Ensembl Fungi

Performing MSA with MAFFT and trimming the alignment:

```
# Perform MSA with MAFFT
aln_fungi <- align_sequences(busco_seqs_fungi, threads = 4)

# Trim alignment to remove columns with >50% of gaps
aln_fungi_trimmed <- lapply(aln_fungi, trim_alignment, max_gap = 0.5)
```

Now, let's infer a species tree using IQ-TREE2.

```
outgroup <- "aphanomyces.astaci,aphanomyces.invadans,globisporangium.ultimum"
trees_fungi <- infer_species_tree(aln_fungi_trimmed, outgroup, threads = 4)
```

Finally, for comparative reasons, we will also infer a single tree from a concatenated multiple sequence alignment.

```
# Concatenate alignments
aln_fungi_conc <- Reduce(xscat, aln_fungi_trimmed)
names(aln_fungi_conc) <- names(aln_fungi_trimmed[[1]])

# Infer tree from concatenated alignment
tree_fungi_conc <- infer_species_tree(
    list(conc = aln_fungi_conc),
    outgroup, threads = 4
)
```

Combining the trees and saving them:

```
# Combine trees
fungi_busco_trees <- c(
    tree_fungi_conc, trees_fungi
)

save(
    fungi_busco_trees, compress = "xz",
    file = here("products", "result_files", "trees", "fungi_busco_trees.rda")
)
```

4.2.2 Ensembl Plants

Here, because no BUSCO gene is present in all species, we will only infer a single tree from concatenated alignments.

```
# Perform MSA with MAFFT
aln_plants <- align_sequences(plants_busco_seqs, threads = 4)
```

```r
# Trim alignment to remove columns with >50% of gaps
aln_plants_trimmed <- lapply(aln_plants, trim_alignment, max_gap = 0.5)
```

Finally, let's infer a species tree from a concatenated alignment. As outgroups, we're going to use *Chondrus crispus*, *Galdieria sulphuraria*, and *Cyanidioschyzon merolae*.

```r
outgroup <- "chondrus.crispus,galdieria.sulphuraria,cyanidioschyzon.merolae"

# Concatenate alignments
aln_plants_conc <- concatenate_alignments(aln_plants_trimmed)

# Infer tree from concatenated alignment
plants_busco_trees <- infer_species_tree(
    list(conc = aln_plants_conc),
    outgroup, threads = 4
)

# Save tree
save(
    plants_busco_trees, compress = "xz",
    file = here("products", "result_files", "trees", "plants_busco_trees.rda")
)
```

4.2.3 Ensembl Protists

For this instance, two BUSCO genes were conserved across all species, so we will infer trees for each family + a tree from a concatenated alignment.

```r
# Perform MSA with MAFFT
aln_protists <- align_sequences(protists_busco_seqs, threads = 4)

# Trim alignment to remove columns with >50% of gaps
aln_protists_trimmed <- lapply(aln_protists, trim_alignment, max_gap = 0.5)
```

Now, let's infer species trees. As outgroup, we will use Fornicata (*Giardia lamblia*) based on this paper.

```r
outgroup <- "giardia.lamblia"

# Path 1: a tree per BUSCO gene
protists_trees1 <- infer_species_tree(
    aln_protists_trimmed, outgroup, threads = 4
)

# Path 2: a single tree from a concatenated alignment
protists_trees2 <- infer_species_tree(
    list(conc = concatenate_alignments(aln_protists_trimmed)),
    outgroup, threads = 6
)
```

```
# Combine trees and save them
protists_busco_trees <- c(protists_trees1, protists_trees2)

save(
    protists_busco_trees, compress = "xz",
    file = here("products", "result_files", "trees", "protists_busco_trees.rda")
)
```

However, even though we specified *Giardia lamblia*, IQ-TREE2 placed it as an ingroup. This suggests that, based on our data (BUSCO sequences), *Giardia lamblia* may not be a good outgroup.

Since protists are not actually a real phylogenetic group (not monophyletic), instead of digging deeper into the real phylogeny of the group and searching for a proper outgroup, we will simply use this phylogeny but acknowledging that it may not be completely accurate.

4.2.4 Ensembl Metazoa

For this instance, two BUSCO genes were conserved across all species, so we will infer trees for each family + a tree from a concatenated alignment.

```
# Perform MSA with MAFFT
aln_metazoa <- align_sequences(metazoa_busco_seqs, threads = 4)

# Trim alignment to remove columns with >50% of gaps
aln_metazoa_trimmed <- lapply(aln_metazoa, trim_alignment, max_gap = 0.5)
```

Now, let's infer a species tree. As outgroup, we will use the ctenophore *Mnemiopsis leidyi*.

```
outgroup <- "mnemiopsis.leidyi"

# Get a single tree from a concatenated alignment
metazoa_busco_trees <- infer_species_tree(
    list(conc = concatenate_alignments(aln_metazoa_trimmed)),
    outgroup, threads = 6
)

# Save tree
save(
    metazoa_busco_trees, compress = "xz",
    file = here("products", "result_files", "trees", "metazoa_busco_trees.rda")
)
```

4.2.5 Ensembl Vertebrates

For this instance, no BUSCO gene was conserved in all species. Thus, we will infer a single tree from a concatenated alignment of ten representative BUSCOs.

```
# Perform MSA with MAFFT
aln_vertebrates <- align_sequences(vertebrates_busco_seqs, threads = 4)
```

```r
# Trim alignment to remove columns with >50% of gaps
aln_vertebrates_trimmed <- lapply(aln_vertebrates, trim_alignment, max_gap = 0.5)
```

Now, let's infer a species tree. As outgroup, we will use the yeast *Saccharomyces cerevisiae*.

```r
outgroup <- "saccharomyces.cerevisiae"

# Get a single tree from a concatenated alignment
vertebrates_busco_trees <- infer_species_tree(
    list(conc = concatenate_alignments(aln_vertebrates_trimmed)),
    outgroup, threads = 6
)

# Save tree
save(
    vertebrates_busco_trees, compress = "xz",
    file = here("products", "result_files", "trees", "vertebrates_busco_trees.rda")
)
```

5 Obtaining BUSCO scores

Finally, since we ran BUSCO to obtain single-copy gene families, we will also use BUSCO's output to explore gene space completeness across species in Ensembl instances.

```r
# Read BUSCO completeness stats
## Ensembl Fungi
fungi_busco_scores <- read_busco(
    "~/Downloads/busco_fungi/ensembl_fungi"
)

## Ensembl Plants
plants_busco_scores <- read_busco(
    "~/Downloads/busco_plants/ensemblplants"
)

## Ensembl Protists
protists_busco_scores <- read_busco(
    "~/Downloads/busco_protists/ensemblprotists"
)

## Ensembl Metazoa
metazoa_busco_scores <- read_busco(
    "~/Downloads/busco_metazoa/ensemblmetazoa"
)

## Ensembl Vertebrates
vertebrates_busco_scores <- read_busco(
    "~/Downloads/busco_vertebrates/ensemblvertebrates"
)
```

```
# Save files
save(
    fungi_busco_scores, compress = "xz",
    file = here(
        "products", "result_files", "busco_scores", "fungi_busco_scores.rda"
    )
)

save(
    plants_busco_scores, compress = "xz",
    file = here(
        "products", "result_files", "busco_scores", "plants_busco_scores.rda"
    )
)

save(
    protists_busco_scores, compress = "xz",
    file = here(
        "products", "result_files", "busco_scores", "protists_busco_scores.rda"
    )
)

save(
    metazoa_busco_scores, compress = "xz",
    file = here(
        "products", "result_files", "busco_scores", "metazoa_busco_scores.rda"
    )
)

save(
    vertebrates_busco_scores, compress = "xz",
    file = here(
        "products", "result_files", "busco_scores", "vertebrates_busco_scores.rda"
    )
)
```

Session info

This document was created under the following conditions:

```
## - Session info ---------------------------------------------------------------
##  setting  value
##  version  R version 4.3.2 (2023-10-31)
##  os       Ubuntu 22.04.3 LTS
##  system   x86_64, linux-gnu
##  ui       X11
##  language (EN)
##  collate  en_US.UTF-8
##  ctype    en_US.UTF-8
```

```
##  tz       Europe/Brussels
##  date     2024-02-27
##  pandoc   3.1.1 @ /usr/lib/rstudio/resources/app/bin/quarto/bin/tools/ (via rmarkdown)
##
## - Packages -----------------------------------------------------------------
##  package          * version     date (UTC) lib source
##  AnnotationDbi      1.64.1      2023-11-03 [1] Bioconductor
##  ape                5.7-1       2023-03-13 [1] CRAN (R 4.3.2)
##  aplot              0.2.2       2023-10-06 [1] CRAN (R 4.3.2)
##  beeswarm           0.4.0       2021-06-01 [1] CRAN (R 4.3.2)
##  Biobase            2.62.0      2023-10-24 [1] Bioconductor
##  BiocFileCache      2.10.1      2023-10-26 [1] Bioconductor
##  BiocGenerics     * 0.48.1      2023-11-01 [1] Bioconductor
##  BiocManager        1.30.22     2023-08-08 [1] CRAN (R 4.3.2)
##  BiocStyle        * 2.30.0      2023-10-24 [1] Bioconductor
##  biomaRt          * 2.58.2      2024-01-30 [1] Bioconductor 3.18 (R 4.3.2)
##  Biostrings       * 2.70.2      2024-01-28 [1] Bioconductor 3.18 (R 4.3.2)
##  bit                4.0.5       2022-11-15 [1] CRAN (R 4.3.2)
##  bit64              4.0.5       2020-08-30 [1] CRAN (R 4.3.2)
##  bitops             1.0-7       2021-04-24 [1] CRAN (R 4.3.2)
##  blob               1.2.4       2023-03-17 [1] CRAN (R 4.3.2)
##  bold               1.3.0       2023-05-02 [1] CRAN (R 4.3.2)
##  bookdown           0.37        2023-12-01 [1] CRAN (R 4.3.2)
##  cachem             1.0.8       2023-05-01 [1] CRAN (R 4.3.2)
##  cli                3.6.2       2023-12-11 [1] CRAN (R 4.3.2)
##  codetools          0.2-19      2023-02-01 [4] CRAN (R 4.2.2)
##  cogeqc           * 1.6.2       2024-01-14 [1] Bioconductor 3.18 (R 4.3.2)
##  colorspace         2.1-0       2023-01-23 [1] CRAN (R 4.3.2)
##  conditionz         0.1.0       2019-04-24 [1] CRAN (R 4.3.2)
##  crayon             1.5.2       2022-09-29 [1] CRAN (R 4.3.2)
##  crul               1.4.0       2023-05-17 [1] CRAN (R 4.3.2)
##  curl               5.2.0       2023-12-08 [1] CRAN (R 4.3.2)
##  data.table         1.15.0      2024-01-30 [1] CRAN (R 4.3.2)
##  DBI                1.2.1       2024-01-12 [1] CRAN (R 4.3.2)
##  dbplyr             2.4.0       2023-10-26 [1] CRAN (R 4.3.2)
##  digest             0.6.34      2024-01-11 [1] CRAN (R 4.3.2)
##  dplyr            * 1.1.4       2023-11-17 [1] CRAN (R 4.3.2)
##  evaluate           0.23        2023-11-01 [1] CRAN (R 4.3.2)
##  fansi              1.0.6       2023-12-08 [1] CRAN (R 4.3.2)
##  fastmap            1.1.1       2023-02-24 [1] CRAN (R 4.3.2)
##  filelock           1.0.3       2023-12-11 [1] CRAN (R 4.3.2)
##  forcats          * 1.0.0       2023-01-29 [1] CRAN (R 4.3.2)
##  foreach            1.5.2       2022-02-02 [1] CRAN (R 4.3.2)
##  fs                 1.6.3       2023-07-20 [1] CRAN (R 4.3.2)
##  generics           0.1.3       2022-07-05 [1] CRAN (R 4.3.2)
##  GenomeInfoDb     * 1.38.6      2024-02-08 [1] Bioconductor 3.18 (R 4.3.2)
##  GenomeInfoDbData   1.2.11      2023-12-21 [1] Bioconductor
##  ggbeeswarm         0.7.2       2023-04-29 [1] CRAN (R 4.3.2)
##  ggfun              0.1.4       2024-01-19 [1] CRAN (R 4.3.2)
##  ggplot2          * 3.4.4       2023-10-12 [1] CRAN (R 4.3.2)
##  ggplotify          0.1.2       2023-08-09 [1] CRAN (R 4.3.2)
```

```
##  ggtree            3.10.0     2023-10-24 [1] Bioconductor
##  glue              1.7.0      2024-01-09 [1] CRAN (R 4.3.2)
##  gridGraphics      0.5-1      2020-12-13 [1] CRAN (R 4.3.2)
##  gtable            0.3.4      2023-08-21 [1] CRAN (R 4.3.2)
##  here            * 1.0.1      2020-12-13 [1] CRAN (R 4.3.2)
##  Herper          * 1.12.0     2023-10-24 [1] Bioconductor
##  hms               1.1.3      2023-03-21 [1] CRAN (R 4.3.2)
##  htmltools         0.5.7      2023-11-03 [1] CRAN (R 4.3.2)
##  httpcode          0.3.0      2020-04-10 [1] CRAN (R 4.3.2)
##  httr              1.4.7      2023-08-15 [1] CRAN (R 4.3.2)
##  igraph            2.0.1.1    2024-01-30 [1] CRAN (R 4.3.2)
##  IRanges         * 2.36.0     2023-10-24 [1] Bioconductor
##  iterators         1.0.14     2022-02-05 [1] CRAN (R 4.3.2)
##  jsonlite          1.8.8      2023-12-04 [1] CRAN (R 4.3.2)
##  KEGGREST          1.42.0     2023-10-24 [1] Bioconductor
##  knitr             1.45       2023-10-30 [1] CRAN (R 4.3.2)
##  lattice           0.22-5     2023-10-24 [4] CRAN (R 4.3.1)
##  lazyeval          0.2.2      2019-03-15 [1] CRAN (R 4.3.2)
##  lifecycle         1.0.4      2023-11-07 [1] CRAN (R 4.3.2)
##  lubridate       * 1.9.3      2023-09-27 [1] CRAN (R 4.3.2)
##  magrittr          2.0.3      2022-03-30 [1] CRAN (R 4.3.2)
##  Matrix            1.6-3      2023-11-14 [4] CRAN (R 4.3.2)
##  memoise           2.0.1      2021-11-26 [1] CRAN (R 4.3.2)
##  munsell           0.5.0      2018-06-12 [1] CRAN (R 4.3.2)
##  nlme              3.1-163    2023-08-09 [4] CRAN (R 4.3.1)
##  patchwork         1.2.0      2024-01-08 [1] CRAN (R 4.3.2)
##  pillar            1.9.0      2023-03-22 [1] CRAN (R 4.3.2)
##  pkgconfig         2.0.3      2019-09-22 [1] CRAN (R 4.3.2)
##  plyr              1.8.9      2023-10-02 [1] CRAN (R 4.3.2)
##  png               0.1-8      2022-11-29 [1] CRAN (R 4.3.2)
##  prettyunits       1.2.0      2023-09-24 [1] CRAN (R 4.3.2)
##  progress          1.2.3      2023-12-06 [1] CRAN (R 4.3.2)
##  purrr           * 1.0.2      2023-08-10 [1] CRAN (R 4.3.2)
##  R6                2.5.1      2021-08-19 [1] CRAN (R 4.3.2)
##  rappdirs          0.3.3      2021-01-31 [1] CRAN (R 4.3.2)
##  Rcpp              1.0.12     2024-01-09 [1] CRAN (R 4.3.2)
##  RCurl             1.98-1.14  2024-01-09 [1] CRAN (R 4.3.2)
##  readr           * 2.1.5      2024-01-10 [1] CRAN (R 4.3.2)
##  reshape2          1.4.4      2020-04-09 [1] CRAN (R 4.3.2)
##  reticulate      * 1.35.0     2024-01-31 [1] CRAN (R 4.3.2)
##  rjson             0.2.21     2022-01-09 [1] CRAN (R 4.3.2)
##  rlang             1.1.3      2024-01-10 [1] CRAN (R 4.3.2)
##  rmarkdown         2.25       2023-09-18 [1] CRAN (R 4.3.2)
##  rprojroot         2.0.4      2023-11-05 [1] CRAN (R 4.3.2)
##  RSQLite           2.3.5      2024-01-21 [1] CRAN (R 4.3.2)
##  rstudioapi        0.15.0     2023-07-07 [1] CRAN (R 4.3.2)
##  S4Vectors       * 0.40.2     2023-11-23 [1] Bioconductor 3.18 (R 4.3.2)
##  scales            1.3.0      2023-11-28 [1] CRAN (R 4.3.2)
##  sessioninfo       1.2.2      2021-12-06 [1] CRAN (R 4.3.2)
##  stringi           1.8.3      2023-12-11 [1] CRAN (R 4.3.2)
##  stringr         * 1.5.1      2023-11-14 [1] CRAN (R 4.3.2)
```

```
##  taxize          * 0.9.100     2022-04-22 [1] CRAN (R 4.3.2)
##  tibble          * 3.2.1       2023-03-20 [1] CRAN (R 4.3.2)
##  tidyr           * 1.3.1       2024-01-24 [1] CRAN (R 4.3.2)
##  tidyselect        1.2.0       2022-10-10 [1] CRAN (R 4.3.2)
##  tidytree          0.4.6       2023-12-12 [1] CRAN (R 4.3.2)
##  tidyverse       * 2.0.0       2023-02-22 [1] CRAN (R 4.3.2)
##  timechange        0.3.0       2024-01-18 [1] CRAN (R 4.3.2)
##  treeio            1.26.0      2023-10-24 [1] Bioconductor
##  tzdb              0.4.0       2023-05-12 [1] CRAN (R 4.3.2)
##  utf8              1.2.4       2023-10-22 [1] CRAN (R 4.3.2)
##  uuid              1.2-0       2024-01-14 [1] CRAN (R 4.3.2)
##  vctrs             0.6.5       2023-12-01 [1] CRAN (R 4.3.2)
##  vipor             0.4.7       2023-12-18 [1] CRAN (R 4.3.2)
##  withr             3.0.0       2024-01-16 [1] CRAN (R 4.3.2)
##  xfun              0.42        2024-02-08 [1] CRAN (R 4.3.2)
##  XML               3.99-0.16.1 2024-01-22 [1] CRAN (R 4.3.2)
##  xml2              1.3.6       2023-12-04 [1] CRAN (R 4.3.2)
##  XVector         * 0.42.0      2023-10-24 [1] Bioconductor
##  yaml              2.3.8       2023-12-11 [1] CRAN (R 4.3.2)
##  yulab.utils       0.1.4       2024-01-28 [1] CRAN (R 4.3.2)
##  zlibbioc          1.48.0      2023-10-24 [1] Bioconductor
##  zoo               1.8-12      2023-04-13 [1] CRAN (R 4.3.2)
##
##  [1] /home/faalm/R/x86_64-pc-linux-gnu-library/4.3
##  [2] /usr/local/lib/R/site-library
##  [3] /usr/lib/R/site-library
##  [4] /usr/lib/R/library
##
## ------------------------------------------------------------------------------
```

Supplementary Text S2: Identification and classification of duplicated genes in Ensembl and Ensembl Genomes

Fabricio Almeida-Silva [1] ***and Yves Van de Peer*** [1]

[1]VIB-UGent Center for Plant Systems Biology, Ghent University, Ghent, Belgium

27 February 2024

Contents

1 Introduction 2

2 Data loading: species trees and metadata 2

3 Identification and classification of duplicated genes in Ensembl and Ensembl Genomes 3

3.1 Ensembl Fungi 3

3.2 Ensembl Protists 4

3.3 Ensembl Plants. 4

3.4 Ensembl Metazoa 6

3.5 Ensembl (Vertebrates) 8

Session info 10

Supplementary Text S2: Identification and classification of duplicated genes in Ensembl and Ensembl Genomes

1 Introduction

Here, we will describe the code to identify and classify duplicated genes in Ensembl and Ensembl Genomes species using the Bioconductor package *doubletrouble*.

```
library(syntenet)
library(doubletrouble)
library(biomaRt)
library(here)
## here() starts at /home/faalm/Dropbox/package_benchmarks/doubletrouble_paper
library(tidyverse)
## -- Attaching core tidyverse packages ------------------------ tidyverse 2.0.0 --
## v dplyr     1.1.4     v readr     2.1.5
## v forcats   1.0.0     v stringr   1.5.1
## v ggplot2   3.4.4     v tibble    3.2.1
## v lubridate 1.9.3     v tidyr     1.3.1
## v purrr     1.0.2
## -- Conflicts ------------------------------------------ tidyverse_conflicts() --
## x dplyr::filter() masks stats::filter()
## x dplyr::lag()    masks stats::lag()
## x dplyr::select() masks biomaRt::select()
## i Use the conflicted package (<http://conflicted.r-lib.org/>) to force all conflicts to become errors

set.seed(123) # for reproducibility
options(timeout = 1e10) # to allow download of big files

# Load helper functions
source(here("code", "utils.R"))
```

2 Data loading: species trees and metadata

Here, we will load the data frames of species metadata and `phylo` objects with species trees for each Ensembl instance.

```
# Load metadata
load(here("products", "result_files", "metadata_all.rda"))
names(metadata_all)
## [1] "fungi"    "plants"   "metazoa"  "protists" "ensembl"

# Load trees
load(here("products", "result_files", "trees", "fungi_busco_trees.rda"))
load(here("products", "result_files", "trees", "plants_busco_trees.rda"))
load(here("products", "result_files", "trees", "metazoa_busco_trees.rda"))
load(here("products", "result_files", "trees", "vertebrates_busco_trees.rda"))
load(here("products", "result_files", "trees", "protists_busco_trees.rda"))
```

3 Identification and classification of duplicated genes in Ensembl and Ensembl Genomes

Now, let's use *doubletrouble* to identify duplicated genes and classify them using the Ensembl and Ensembl Genomes data sets. Here, to avoid code repetition and optimize memory usage, we will use the wrapper function `ensembl2duplicates()` (in the file *utils.R*). For each species in the metadata data frame, this function:

1. Retrieves whole-genome protein sequences (`AAStringSet`) and gene annotation (`GRanges`) from an Ensembl instance;
2. Filters the `AAStringSet` object to include only the longest protein for each gene (i.e., the translated sequence of the primary transcript);
3. Processes the sequences and annotation with `syntenet::process_input()`;
4. Identifies the paranome with `syntenet::run_diamond()` + identifies orthologs between the query species and an outgroup (optional);
5. Classifies paralogs by duplication modes.

3.1 Ensembl Fungi

First, let's create a data frame with species and their outgroups. Here, we will use the basidiomycete *Cryptococcus neoformans* as outgroup for Ascomycota species, and the oomycete *Aphanomyces astaci* as outgroup for Basidiomycota species.

```
col_dir <- here("products", "result_files", "collinearity", "fungi")
if(!dir.exists(col_dir)) { dir.create(col_dir, recursive = TRUE) }

# Create data frame of query species and outgroup
fungi_outgroups <- metadata_all$fungi |>
    filter(phylum != "Oomycota") |>
    mutate(
        query = species,
        outgroup = case_when(
            phylum == "Ascomycota" ~ "cryptococcus_neoformans",
            TRUE ~ "aphanomyces_astaci"
        )
    ) |>
    select(query, outgroup)

# Identifying and classifying paralogs
fungi_duplicates <- ensembl2duplicates(
    metadata_all$fungi, ensembl = "fungi",
    outgroups = fungi_outgroups,
    collinearity_dir = col_dir
)

# Classify genes into unique duplication modes
fungi_duplicates_unique <- classify_genes(fungi_duplicates)

# Save classification results
```

```
## Duplicate pairs
save(
    fungi_duplicates,
    file = here("products", "result_files", "fungi_duplicates.rda"),
    compress = "xz"
)

## Duplicated genes (unique duplication modes)
save(
    fungi_duplicates_unique,
    file = here("products", "result_files", "fungi_duplicates_unique.rda"),
    compress = "xz"
)
```

3.2 Ensembl Protists

Since protists are not a real (i.e., monophyletic) group, defining an outgroup species is very problematic. For this reason, we will classify duplicates using the *standard* classification scheme here.

```
col_dir <- here("products", "result_files", "collinearity", "protists")
if(!dir.exists(col_dir)) { dir.create(col_dir, recursive = TRUE) }

# Identifying and classifying paralogs
protists_duplicates <- ensembl2duplicates(
    metadata_all$protists, ensembl = "protists", collinearity_dir = col_dir
)

# Classify genes into unique duplication modes
protists_duplicates_unique <- classify_genes(protists_duplicates)

# Save classification results
## Duplicate pairs
save(
    protists_duplicates,
    file = here("products", "result_files", "protists_duplicates.rda"),
    compress = "xz"
)

## Duplicated genes (unique duplication modes)
save(
    protists_duplicates_unique,
    file = here("products", "result_files", "protists_duplicates_unique.rda"),
    compress = "xz"
)
```

3.3 Ensembl Plants

Here, we will use different outgroups for different branches of the tree. The clades and outgroups are:

1. Angiosperms: *Amborella trichopoda* as outgroup.
2. *Amborella trichopoda* and *Nymphaea colorata*: *Chara braunii* as outgroup.
3. *Selaginella moellendorffii*, *Chara braunii*, *Marchantia polymorpha*, *Physcomitrium patens*: *Chlamydomonas reinhardtii* as outgroup.
4. *Chlamydomonas reinhardtii* and *Ostreococcus lucimarinus*: *Galdieria sulphuraria* as outgroup
5. Rhodophyta algae: no outgroup.

```
# Create data frame of query species and outgroup
angiosperms <- metadata_all$plants |>
    filter(
        phylum == "Streptophyta",
        !order %in% c(
            "Charales", "Selaginellales", "Funariales",
            "Marchantiales", "Nymphaeales"
        )
    ) |>
    pull(species)

ana <- c("amborella_trichopoda", "nymphaea_colorata")
bryophytes <- c(
    "selaginella_moellendorffii", "chara_braunii",
    "marchantia_polymorpha", "physcomitrium_patens"
)
chlorophyta <- c("chlamydomonas_reinhardtii", "ostreococcus_lucimarinus")

plants_outgroups <- metadata_all$plants |>
    filter(phylum != "Rhodophyta") |>
    mutate(
        query = species,
        outgroup = case_when(
            species %in% angiosperms ~ "amborella_trichopoda",
            species %in% ana ~ "chara_braunii",
            species %in% bryophytes ~ "chlamydomonas_reinhardtii",
            species %in% chlorophyta ~ "galdieria_sulphuraria"
        )
    ) |>
    select(query, outgroup)
```

Identifying and classifying duplicates:

```
col_dir <- here("products", "result_files", "collinearity", "plants")
if(!dir.exists(col_dir)) { dir.create(col_dir, recursive = TRUE) }

# Identifying and classifying paralogs
plants_duplicates <- ensembl2duplicates(
    metadata_all$plants, ensembl = "plants",
    outgroups = plants_outgroups,
    collinearity_dir = col_dir,
    threads = 4
```

```
)

# Classify genes into unique duplication modes
plants_duplicates_unique <- classify_genes(plants_duplicates)

# Save classification results
## Duplicate pairs
save(
    plants_duplicates,
    file = here("products", "result_files", "plants_duplicates.rda"),
    compress = "xz"
)

## Duplicated genes (unique duplication modes)
save(
    plants_duplicates_unique,
    file = here("products", "result_files", "plants_duplicates_unique.rda"),
    compress = "xz"
)
```

3.4 Ensembl Metazoa

Here, we will use different outgroups for different branches of the tree. The clades and outgroups are:

1. Arthropoda: *Hypsibius exemplaris* (Tardigrada) as outgroup.
2. Tardigrada, Acanthocephala, and Rotifera: *Brugia malayi* (Nematoda) as outgroup.
3. Nematoda: *Priapulus caudatus* (Priapulida) as outgroup
4. Priapulida, Echinodermata, Chordata, and Hemichordata: *Hofstenia miamia* (Xenacoelomorpha) as outgroup.
5. Xenacoelomorpha: *Actinia tenebrosa* (Cnidaria) as outgroup.
6. Cnidaria and Placozoa: *Amphimedon queenslandica* (Porifera) as outgroup.
7. Porifera: *Mnemiopsis leidyi* (Ctenophora) as outgroup.
8. Brachiopoda: *Haliotis rufescens* (Mollusca) as outgroup.
9. Mollusca, Annelida, and Platyhelminthes: *Adineta vaga* (Rotifera) as outgroup.

```
# Create data frame of query species and outgroup
by_phylum <- function(df, taxon) {
    return(
        df |>
            dplyr::filter(phylum == taxon) |>
            dplyr::pull(species)
    )
}

arthropoda <- by_phylum(metadata_all$metazoa, "Arthropoda")
tardigrada <- by_phylum(metadata_all$metazoa, "Tardigrada")
```

```
nematoda <- by_phylum(metadata_all$metazoa, "Nematoda")
priapulida <- by_phylum(metadata_all$metazoa, "Priapulida")
xenacoelomorpha <- by_phylum(metadata_all$metazoa, "Xenacoelomorpha")
cnidaria <- by_phylum(metadata_all$metazoa, "Cnidaria")
placozoa <- by_phylum(metadata_all$metazoa, "Placozoa")
porifera <- by_phylum(metadata_all$metazoa, "Porifera")
brachiopoda <- by_phylum(metadata_all$metazoa, "Brachiopoda")
mollusca <- by_phylum(metadata_all$metazoa, "Mollusca")
echinodermata <- by_phylum(metadata_all$metazoa, "Echinodermata")
annelida <- by_phylum(metadata_all$metazoa, "Annelida")
platyhelminthes <- by_phylum(metadata_all$metazoa, "Platyhelminthes")
acanthocephala <- by_phylum(metadata_all$metazoa, "Acanthocephala")
chordata <- by_phylum(metadata_all$metazoa, "Chordata")
hemichordata <- by_phylum(metadata_all$metazoa, "Hemichordata")
rotifera <- by_phylum(metadata_all$metazoa, "Rotifera")

metazoa_outgroups <- metadata_all$metazoa |>
    filter(phylum != "Ctenophora") |>
    mutate(
        query = species,
        outgroup = case_when(
            species %in% arthropoda ~ "hypsibius_exemplaris_gca002082055v1",
            species %in% c(tardigrada, acanthocephala, rotifera) ~ "brugia_malayi",
            species %in% nematoda ~ "priapulus_caudatus_gca000485595v2",

            species %in% c(priapulida, echinodermata, chordata, hemichordata) ~
                "hofstenia_miamia",
            species %in% xenacoelomorpha ~ "actinia_tenebrosa_gca009602425v1",
            species %in% c(cnidaria, placozoa) ~
                "amphimedon_queenslandica_gca000090795v2rs",
            species %in% porifera ~ "mnemiopsis_leidyi",
            species %in% brachiopoda ~ "haliotis_rufescens_gca023055435v1rs",
            species %in% c(mollusca, annelida, platyhelminthes) ~ "adineta_vaga"
        )
    ) |>
    select(query, outgroup)
```

Identifying and classifying duplicates:

```
col_dir <- here("products", "result_files", "collinearity", "metazoa")
if(!dir.exists(col_dir)) { dir.create(col_dir, recursive = TRUE) }

# Identifying and classifying paralogs
metazoa_duplicates <- ensembl2duplicates(
    metadata = metadata_all$metazoa,
    ensembl = "metazoa",
    outgroups = metazoa_outgroups,
    collinearity_dir = col_dir,
    threads = 4
)
```

```r
# Classify genes into unique duplication modes
metazoa_duplicates_unique <- classify_genes(metazoa_duplicates)

# Save classification results
## Duplicate pairs
save(
    metazoa_duplicates,
    file = here("products", "result_files", "metazoa_duplicates.rda"),
    compress = "xz"
)

## Duplicated genes (unique duplication modes)
save(
    metazoa_duplicates_unique,
    file = here("products", "result_files", "metazoa_duplicates_unique.rda"),
    compress = "xz"
)
```

3.5 Ensembl (Vertebrates)

Here, we will use the following outgroups per taxa:

1. Amniota: *Xenopus tropicalis* (Amphibia) as outgroup;
2. Amphibia: *Latimeria chalumnae* (West Indian Ocean coelacanth)
3. All bony and cartilaginous fish: *Eptatretus burgeri* (hagfish, Agnatha)
4. Agnatha: *Ciona intestinalis* (Tunicata)

```r
# Create a data frame of species and outgroups
amniota <- metadata_all$ensembl |>
    filter(
        class %in% c("Aves", "Mammalia", "Lepidosauria") |
            order %in% c("Testudines", "Crocodylia")
    ) |>
    pull(species)

amphibia <- metadata_all$ensembl |>
    filter(class == "Amphibia") |>
    pull(species)

fish <- metadata_all$ensembl |>
    filter(
        class %in% c("Actinopteri", "Chondrichthyes", "Cladistia") |
            order == "Coelacanthiformes"
    ) |>
    pull(species)

agnatha <- metadata_all$ensembl |>
    filter(
        class %in% c("Myxini", "Hyperoartia")
    ) |>
    pull(species)
```

```
ensembl_outgroups <- metadata_all$ensembl |>
    filter(!phylum %in% c("Nematoda", "Arthropoda", "Ascomycota")) |>
    mutate(
        query = species,
        outgroup = case_when(
            species %in% amniota ~ "xenopus_tropicalis",
            species %in% amphibia ~ "latimeria_chalumnae",
            species %in% fish ~ "eptatretus_burgeri",
            species %in% agnatha ~ "ciona_intestinalis"
        )
    ) |>
    select(query, outgroup) |>
    filter(!is.na(outgroup))
```

Identifying and classifying duplicates:

```
col_dir <- here("products", "result_files", "collinearity", "vertebrates")
if(!dir.exists(col_dir)) { dir.create(col_dir, recursive = TRUE) }

# Identifying and classifying paralogs
vertebrates_duplicates <- ensembl2duplicates(
    meta,
    ensembl = "ensembl",
    outgroups = ensembl_outgroups,
    collinearity_dir = col_dir,
    tsv_dir = "~/Documents/vertebrates_duplicates", # delete later
    threads = 4
)

# Classify genes into unique duplication modes
vertebrates_duplicates_unique <- classify_genes(vertebrates_duplicates)

# Save classification results
## Duplicate pairs
save(
    vertebrates_duplicates,
    file = here("products", "result_files", "vertebrates_duplicates.rda"),
    compress = "xz"
)

## Duplicated genes (unique duplication modes)
save(
    vertebrates_duplicates_unique,
    file = here("products", "result_files", "vertebrates_duplicates_unique.rda"),
    compress = "xz"
)
```

Session info

This document was created under the following conditions:

```
## - Session info ---------------------------------------------------------------
##  setting  value
##  version  R version 4.3.2 (2023-10-31)
##  os       Ubuntu 22.04.3 LTS
##  system   x86_64, linux-gnu
##  ui       X11
##  language (EN)
##  collate  en_US.UTF-8
##  ctype    en_US.UTF-8
##  tz       Europe/Brussels
##  date     2024-02-27
##  pandoc   3.1.1 @ /usr/lib/rstudio/resources/app/bin/quarto/bin/tools/ (via rmarkdown)
##
## - Packages -------------------------------------------------------------------
##  package              * version     date (UTC) lib source
##  abind                  1.4-5       2016-07-21 [1] CRAN (R 4.3.2)
##  ade4                   1.7-22      2023-02-06 [1] CRAN (R 4.3.2)
##  AnnotationDbi          1.64.1      2023-11-03 [1] Bioconductor
##  ape                    5.7-1       2023-03-13 [1] CRAN (R 4.3.2)
##  Biobase                2.62.0      2023-10-24 [1] Bioconductor
##  BiocFileCache          2.10.1      2023-10-26 [1] Bioconductor
##  BiocGenerics           0.48.1      2023-11-01 [1] Bioconductor
##  BiocIO                 1.12.0      2023-10-24 [1] Bioconductor
##  BiocManager            1.30.22     2023-08-08 [1] CRAN (R 4.3.2)
##  BiocParallel           1.37.0      2024-01-19 [1] Github (Bioconductor/BiocParallel@79a1b2d)
##  BiocStyle            * 2.30.0      2023-10-24 [1] Bioconductor
##  biomaRt              * 2.58.2      2024-01-30 [1] Bioconductor 3.18 (R 4.3.2)
##  Biostrings             2.70.2      2024-01-28 [1] Bioconductor 3.18 (R 4.3.2)
##  bit                    4.0.5       2022-11-15 [1] CRAN (R 4.3.2)
##  bit64                  4.0.5       2020-08-30 [1] CRAN (R 4.3.2)
##  bitops                 1.0-7       2021-04-24 [1] CRAN (R 4.3.2)
##  blob                   1.2.4       2023-03-17 [1] CRAN (R 4.3.2)
##  bookdown               0.37        2023-12-01 [1] CRAN (R 4.3.2)
##  cachem                 1.0.8       2023-05-01 [1] CRAN (R 4.3.2)
##  cli                    3.6.2       2023-12-11 [1] CRAN (R 4.3.2)
##  coda                   0.19-4.1    2024-01-31 [1] CRAN (R 4.3.2)
##  codetools              0.2-19      2023-02-01 [4] CRAN (R 4.2.2)
##  colorspace             2.1-0       2023-01-23 [1] CRAN (R 4.3.2)
##  crayon                 1.5.2       2022-09-29 [1] CRAN (R 4.3.2)
##  curl                   5.2.0       2023-12-08 [1] CRAN (R 4.3.2)
##  DBI                    1.2.1       2024-01-12 [1] CRAN (R 4.3.2)
##  dbplyr                 2.4.0       2023-10-26 [1] CRAN (R 4.3.2)
##  DelayedArray           0.28.0      2023-10-24 [1] Bioconductor
##  digest                 0.6.34      2024-01-11 [1] CRAN (R 4.3.2)
##  doParallel             1.0.17      2022-02-07 [1] CRAN (R 4.3.2)
##  doubletrouble        * 1.3.4       2024-02-05 [1] Bioconductor
##  dplyr                * 1.1.4       2023-11-17 [1] CRAN (R 4.3.2)
```

```
## evaluate              0.23     2023-11-01 [1] CRAN (R 4.3.2)
## fansi                 1.0.6    2023-12-08 [1] CRAN (R 4.3.2)
## fastmap               1.1.1    2023-02-24 [1] CRAN (R 4.3.2)
## filelock              1.0.3    2023-12-11 [1] CRAN (R 4.3.2)
## forcats             * 1.0.0    2023-01-29 [1] CRAN (R 4.3.2)
## foreach               1.5.2    2022-02-02 [1] CRAN (R 4.3.2)
## generics              0.1.3    2022-07-05 [1] CRAN (R 4.3.2)
## GenomeInfoDb          1.38.6   2024-02-08 [1] Bioconductor 3.18 (R 4.3.2)
## GenomeInfoDbData      1.2.11   2023-12-21 [1] Bioconductor
## GenomicAlignments     1.38.2   2024-01-16 [1] Bioconductor 3.18 (R 4.3.2)
## GenomicFeatures       1.54.3   2024-01-31 [1] Bioconductor 3.18 (R 4.3.2)
## GenomicRanges         1.54.1   2023-10-29 [1] Bioconductor
## ggnetwork             0.5.13   2024-02-14 [1] CRAN (R 4.3.2)
## ggplot2             * 3.4.4    2023-10-12 [1] CRAN (R 4.3.2)
## glue                  1.7.0    2024-01-09 [1] CRAN (R 4.3.2)
## gtable                0.3.4    2023-08-21 [1] CRAN (R 4.3.2)
## here                * 1.0.1    2020-12-13 [1] CRAN (R 4.3.2)
## hms                   1.1.3    2023-03-21 [1] CRAN (R 4.3.2)
## htmltools             0.5.7    2023-11-03 [1] CRAN (R 4.3.2)
## htmlwidgets           1.6.4    2023-12-06 [1] CRAN (R 4.3.2)
## httr                  1.4.7    2023-08-15 [1] CRAN (R 4.3.2)
## igraph                2.0.1.1  2024-01-30 [1] CRAN (R 4.3.2)
## intergraph            2.0-4    2024-02-01 [1] CRAN (R 4.3.2)
## IRanges               2.36.0   2023-10-24 [1] Bioconductor
## iterators             1.0.14   2022-02-05 [1] CRAN (R 4.3.2)
## KEGGREST              1.42.0   2023-10-24 [1] Bioconductor
## knitr                 1.45     2023-10-30 [1] CRAN (R 4.3.2)
## lattice               0.22-5   2023-10-24 [4] CRAN (R 4.3.1)
## lifecycle             1.0.4    2023-11-07 [1] CRAN (R 4.3.2)
## lubridate           * 1.9.3    2023-09-27 [1] CRAN (R 4.3.2)
## magrittr              2.0.3    2022-03-30 [1] CRAN (R 4.3.2)
## MASS                  7.3-60   2023-05-04 [4] CRAN (R 4.3.1)
## Matrix                1.6-3    2023-11-14 [4] CRAN (R 4.3.2)
## MatrixGenerics        1.14.0   2023-10-24 [1] Bioconductor
## matrixStats           1.2.0    2023-12-11 [1] CRAN (R 4.3.2)
## mclust                6.0.1    2023-11-15 [1] CRAN (R 4.3.2)
## memoise               2.0.1    2021-11-26 [1] CRAN (R 4.3.2)
## MSA2dist              1.6.0    2023-10-24 [1] Bioconductor
## munsell               0.5.0    2018-06-12 [1] CRAN (R 4.3.2)
## network               1.18.2   2023-12-05 [1] CRAN (R 4.3.2)
## networkD3             0.4      2017-03-18 [1] CRAN (R 4.3.2)
## nlme                  3.1-163  2023-08-09 [4] CRAN (R 4.3.1)
## pheatmap              1.0.12   2019-01-04 [1] CRAN (R 4.3.2)
## pillar                1.9.0    2023-03-22 [1] CRAN (R 4.3.2)
## pkgconfig             2.0.3    2019-09-22 [1] CRAN (R 4.3.2)
## png                   0.1-8    2022-11-29 [1] CRAN (R 4.3.2)
## prettyunits           1.2.0    2023-09-24 [1] CRAN (R 4.3.2)
## progress              1.2.3    2023-12-06 [1] CRAN (R 4.3.2)
## purrr               * 1.0.2    2023-08-10 [1] CRAN (R 4.3.2)
## R6                    2.5.1    2021-08-19 [1] CRAN (R 4.3.2)
## rappdirs              0.3.3    2021-01-31 [1] CRAN (R 4.3.2)
```

```
## RColorBrewer            1.1-3       2022-04-03 [1] CRAN (R 4.3.2)
## Rcpp                    1.0.12      2024-01-09 [1] CRAN (R 4.3.2)
## RCurl                   1.98-1.14   2024-01-09 [1] CRAN (R 4.3.2)
## readr                 * 2.1.5       2024-01-10 [1] CRAN (R 4.3.2)
## restfulr                0.0.15      2022-06-16 [1] CRAN (R 4.3.2)
## rjson                   0.2.21      2022-01-09 [1] CRAN (R 4.3.2)
## rlang                   1.1.3       2024-01-10 [1] CRAN (R 4.3.2)
## rmarkdown               2.25        2023-09-18 [1] CRAN (R 4.3.2)
## rprojroot               2.0.4       2023-11-05 [1] CRAN (R 4.3.2)
## Rsamtools               2.18.0      2023-10-24 [1] Bioconductor
## RSQLite                 2.3.5       2024-01-21 [1] CRAN (R 4.3.2)
## rstudioapi              0.15.0      2023-07-07 [1] CRAN (R 4.3.2)
## rtracklayer             1.62.0      2023-10-24 [1] Bioconductor
## S4Arrays                1.2.0       2023-10-24 [1] Bioconductor
## S4Vectors               0.40.2      2023-11-23 [1] Bioconductor 3.18 (R 4.3.2)
## scales                  1.3.0       2023-11-28 [1] CRAN (R 4.3.2)
## seqinr                  4.2-36      2023-12-08 [1] CRAN (R 4.3.2)
## sessioninfo             1.2.2       2021-12-06 [1] CRAN (R 4.3.2)
## SparseArray             1.2.4       2024-02-11 [1] Bioconductor 3.18 (R 4.3.2)
## statnet.common          4.9.0       2023-05-24 [1] CRAN (R 4.3.2)
## stringi                 1.8.3       2023-12-11 [1] CRAN (R 4.3.2)
## stringr               * 1.5.1       2023-11-14 [1] CRAN (R 4.3.2)
## SummarizedExperiment    1.32.0      2023-10-24 [1] Bioconductor
## syntenet              * 1.4.0       2023-10-24 [1] Bioconductor
## tibble                * 3.2.1       2023-03-20 [1] CRAN (R 4.3.2)
## tidyr                 * 1.3.1       2024-01-24 [1] CRAN (R 4.3.2)
## tidyselect              1.2.0       2022-10-10 [1] CRAN (R 4.3.2)
## tidyverse             * 2.0.0       2023-02-22 [1] CRAN (R 4.3.2)
## timechange              0.3.0       2024-01-18 [1] CRAN (R 4.3.2)
## tzdb                    0.4.0       2023-05-12 [1] CRAN (R 4.3.2)
## utf8                    1.2.4       2023-10-22 [1] CRAN (R 4.3.2)
## vctrs                   0.6.5       2023-12-01 [1] CRAN (R 4.3.2)
## withr                   3.0.0       2024-01-16 [1] CRAN (R 4.3.2)
## xfun                    0.42        2024-02-08 [1] CRAN (R 4.3.2)
## XML                     3.99-0.16.1 2024-01-22 [1] CRAN (R 4.3.2)
## xml2                    1.3.6       2023-12-04 [1] CRAN (R 4.3.2)
## XVector                 0.42.0      2023-10-24 [1] Bioconductor
## yaml                    2.3.8       2023-12-11 [1] CRAN (R 4.3.2)
## zlibbioc                1.48.0      2023-10-24 [1] Bioconductor
##
## [1] /home/faalm/R/x86_64-pc-linux-gnu-library/4.3
## [2] /usr/local/lib/R/site-library
## [3] /usr/lib/R/site-library
## [4] /usr/lib/R/library
##
## ------------------------------------------------------------------------------
```

Supplementary Text S3: Calculating substitution rates for selected Ensembl genomes

Fabricio Almeida-Silva [1] ***and Yves Van de Peer*** [1]

[1]VIB-UGent Center for Plant Systems Biology, Ghent University, Ghent, Belgium

27 February 2024

Contents

1 Introduction 2
2 Data loading. 2
3 Calculating substitution rates 2
Session info 3

1 Introduction

Here, we will describe the code to calculate substitution rates for selected genomes in Ensembl and Ensembl Genomes instances using the Bioconductor package *doubletrouble*.

```
library(syntenet)
library(doubletrouble)
library(here)
## here() starts at /home/faalm/Dropbox/package_benchmarks/doubletrouble_paper
library(tidyverse)
## -- Attaching core tidyverse packages ------------------------ tidyverse 2.0.0 --
## v dplyr     1.1.4     v readr     2.1.5
## v forcats   1.0.0     v stringr   1.5.1
## v ggplot2   3.4.4     v tibble    3.2.1
## v lubridate 1.9.3     v tidyr     1.3.1
## v purrr     1.0.2
## -- Conflicts ------------------------------------------ tidyverse_conflicts() --
## x dplyr::filter() masks stats::filter()
## x dplyr::lag()    masks stats::lag()
## i Use the conflicted package (<http://conflicted.r-lib.org/>) to force all conflicts to become errors
library(BiocParallel)

set.seed(123) # for reproducibility
options(timeout = 1e10) # to allow download of big files

# Load helper functions
source(here("code", "utils.R"))
```

2 Data loading

Here, we will load the data frames of species metadata and the lists of duplicated gene pairs for each Ensembl instance.

```
# Load metadata
load(here("products", "result_files", "metadata_all.rda"))
names(metadata_all)
## [1] "fungi"    "plants"   "metazoa"  "protists" "ensembl"

# Load duplicates
load(here("products", "result_files", "fungi_duplicates.rda"))
load(here("products", "result_files", "plants_duplicates.rda"))
```

3 Calculating substitution rates

Next, we will calculate substitution rates (K_a, K_s, and K_a/K_s) for duplicate pairs in all selected species, namely:

1. Three fungi species (*Saccharomyces cerevisiae*, *Candida glabrata*, and *Schizosaccharomyces pombe*).

2. Four legume species (*Glycine max*, *Phaseolus vulgaris*, *Vitis vinifera*, *Selaginella moellendorffii*).

```r
# Fungi - S. cerevisiae, Candida glabrata, and Schizosaccharomyces pombe
## Download CDS
selected_fungi <- c(
    "saccharomyces_cerevisiae", "candida_glabrata", "schizosaccharomyces_pombe"
)
fungi_cds <- get_cds_ensembl(selected_fungi, ensembl = "fungi")

## Calculate substitution rates
fungi_kaks <- pairs2kaks(
    gene_pairs_list = fungi_duplicates[selected_fungi],
    cds = fungi_cds,
    bp_param = BiocParallel::SnowParam(workers = 8)
)

# Plants - Glycine max, Phaseolus vulgaris, Vitis vinifera, and Selaginella moellendorffii
## Download CDS
selected_plants <- c(
    "glycine_max", "phaseolus_vulgaris", "vitis_vinifera",
    "selaginella_moellendorffii"
)

plants_cds <- get_cds_ensembl(selected_plants, ensembl = "plants")

## Calculate substitution rates
plants_duplicates <- plants_duplicates[selected_plants]

plants_kaks <- pairs2kaks(
    gene_pairs_list = plants_duplicates,
    cds = plants_cds,
    bp_param = BiocParallel::SnowParam(workers = 8)
)
```

Saving objects as .rda files:

```r
save(
    fungi_kaks, compress = "xz",
    file = here("products", "result_files", "fungi_kaks.rda")
)

save(
    plants_kaks, compress = "xz",
    file = here("products", "result_files", "plants_kaks.rda")
)
```

Session info

This document was created under the following conditions:

Supplementary Text S3: Calculating substitution rates for selected Ensembl genomes

```
## - Session info ---------------------------------------------------------------
##  setting  value
##  version  R version 4.3.2 (2023-10-31)
##  os       Ubuntu 22.04.3 LTS
##  system   x86_64, linux-gnu
##  ui       X11
##  language (EN)
##  collate  en_US.UTF-8
##  ctype    en_US.UTF-8
##  tz       Europe/Brussels
##  date     2024-02-27
##  pandoc   3.1.1 @ /usr/lib/rstudio/resources/app/bin/quarto/bin/tools/ (via rmarkdown)
##
## - Packages -------------------------------------------------------------------
##  package              * version     date (UTC) lib source
##  abind                  1.4-5       2016-07-21 [1] CRAN (R 4.3.2)
##  ade4                   1.7-22      2023-02-06 [1] CRAN (R 4.3.2)
##  AnnotationDbi          1.64.1      2023-11-03 [1] Bioconductor
##  ape                    5.7-1       2023-03-13 [1] CRAN (R 4.3.2)
##  Biobase                2.62.0      2023-10-24 [1] Bioconductor
##  BiocFileCache          2.10.1      2023-10-26 [1] Bioconductor
##  BiocGenerics           0.48.1      2023-11-01 [1] Bioconductor
##  BiocIO                 1.12.0      2023-10-24 [1] Bioconductor
##  BiocManager            1.30.22     2023-08-08 [1] CRAN (R 4.3.2)
##  BiocParallel         * 1.37.0      2024-01-19 [1] Github (Bioconductor/BiocParallel@79a1b2d)
##  BiocStyle            * 2.30.0      2023-10-24 [1] Bioconductor
##  biomaRt                2.58.2      2024-01-30 [1] Bioconductor 3.18 (R 4.3.2)
##  Biostrings             2.70.2      2024-01-28 [1] Bioconductor 3.18 (R 4.3.2)
##  bit                    4.0.5       2022-11-15 [1] CRAN (R 4.3.2)
##  bit64                  4.0.5       2020-08-30 [1] CRAN (R 4.3.2)
##  bitops                 1.0-7       2021-04-24 [1] CRAN (R 4.3.2)
##  blob                   1.2.4       2023-03-17 [1] CRAN (R 4.3.2)
##  bookdown               0.37        2023-12-01 [1] CRAN (R 4.3.2)
##  cachem                 1.0.8       2023-05-01 [1] CRAN (R 4.3.2)
##  cli                    3.6.2       2023-12-11 [1] CRAN (R 4.3.2)
##  coda                   0.19-4.1    2024-01-31 [1] CRAN (R 4.3.2)
##  codetools              0.2-19      2023-02-01 [4] CRAN (R 4.2.2)
##  colorspace             2.1-0       2023-01-23 [1] CRAN (R 4.3.2)
##  crayon                 1.5.2       2022-09-29 [1] CRAN (R 4.3.2)
##  curl                   5.2.0       2023-12-08 [1] CRAN (R 4.3.2)
##  DBI                    1.2.1       2024-01-12 [1] CRAN (R 4.3.2)
##  dbplyr                 2.4.0       2023-10-26 [1] CRAN (R 4.3.2)
##  DelayedArray           0.28.0      2023-10-24 [1] Bioconductor
##  digest                 0.6.34      2024-01-11 [1] CRAN (R 4.3.2)
##  doParallel             1.0.17      2022-02-07 [1] CRAN (R 4.3.2)
##  doubletrouble        * 1.3.4       2024-02-05 [1] Bioconductor
##  dplyr                * 1.1.4       2023-11-17 [1] CRAN (R 4.3.2)
##  evaluate               0.23        2023-11-01 [1] CRAN (R 4.3.2)
##  fansi                  1.0.6       2023-12-08 [1] CRAN (R 4.3.2)
##  fastmap                1.1.1       2023-02-24 [1] CRAN (R 4.3.2)
##  filelock               1.0.3       2023-12-11 [1] CRAN (R 4.3.2)
```

```
##  forcats             * 1.0.0      2023-01-29 [1] CRAN (R 4.3.2)
##  foreach               1.5.2      2022-02-02 [1] CRAN (R 4.3.2)
##  generics              0.1.3      2022-07-05 [1] CRAN (R 4.3.2)
##  GenomeInfoDb          1.38.6     2024-02-08 [1] Bioconductor 3.18 (R 4.3.2)
##  GenomeInfoDbData      1.2.11     2023-12-21 [1] Bioconductor
##  GenomicAlignments     1.38.2     2024-01-16 [1] Bioconductor 3.18 (R 4.3.2)
##  GenomicFeatures       1.54.3     2024-01-31 [1] Bioconductor 3.18 (R 4.3.2)
##  GenomicRanges         1.54.1     2023-10-29 [1] Bioconductor
##  ggnetwork             0.5.13     2024-02-14 [1] CRAN (R 4.3.2)
##  ggplot2             * 3.4.4      2023-10-12 [1] CRAN (R 4.3.2)
##  glue                  1.7.0      2024-01-09 [1] CRAN (R 4.3.2)
##  gtable                0.3.4      2023-08-21 [1] CRAN (R 4.3.2)
##  here                * 1.0.1      2020-12-13 [1] CRAN (R 4.3.2)
##  hms                   1.1.3      2023-03-21 [1] CRAN (R 4.3.2)
##  htmltools             0.5.7      2023-11-03 [1] CRAN (R 4.3.2)
##  htmlwidgets           1.6.4      2023-12-06 [1] CRAN (R 4.3.2)
##  httr                  1.4.7      2023-08-15 [1] CRAN (R 4.3.2)
##  igraph                2.0.1.1    2024-01-30 [1] CRAN (R 4.3.2)
##  intergraph            2.0-4      2024-02-01 [1] CRAN (R 4.3.2)
##  IRanges               2.36.0     2023-10-24 [1] Bioconductor
##  iterators             1.0.14     2022-02-05 [1] CRAN (R 4.3.2)
##  KEGGREST              1.42.0     2023-10-24 [1] Bioconductor
##  knitr                 1.45       2023-10-30 [1] CRAN (R 4.3.2)
##  lattice               0.22-5     2023-10-24 [4] CRAN (R 4.3.1)
##  lifecycle             1.0.4      2023-11-07 [1] CRAN (R 4.3.2)
##  lubridate           * 1.9.3      2023-09-27 [1] CRAN (R 4.3.2)
##  magrittr              2.0.3      2022-03-30 [1] CRAN (R 4.3.2)
##  MASS                  7.3-60     2023-05-04 [4] CRAN (R 4.3.1)
##  Matrix                1.6-3      2023-11-14 [4] CRAN (R 4.3.2)
##  MatrixGenerics        1.14.0     2023-10-24 [1] Bioconductor
##  matrixStats           1.2.0      2023-12-11 [1] CRAN (R 4.3.2)
##  mclust                6.0.1      2023-11-15 [1] CRAN (R 4.3.2)
##  memoise               2.0.1      2021-11-26 [1] CRAN (R 4.3.2)
##  MSA2dist              1.6.0      2023-10-24 [1] Bioconductor
##  munsell               0.5.0      2018-06-12 [1] CRAN (R 4.3.2)
##  network               1.18.2     2023-12-05 [1] CRAN (R 4.3.2)
##  networkD3             0.4        2017-03-18 [1] CRAN (R 4.3.2)
##  nlme                  3.1-163    2023-08-09 [4] CRAN (R 4.3.1)
##  pheatmap              1.0.12     2019-01-04 [1] CRAN (R 4.3.2)
##  pillar                1.9.0      2023-03-22 [1] CRAN (R 4.3.2)
##  pkgconfig             2.0.3      2019-09-22 [1] CRAN (R 4.3.2)
##  png                   0.1-8      2022-11-29 [1] CRAN (R 4.3.2)
##  prettyunits           1.2.0      2023-09-24 [1] CRAN (R 4.3.2)
##  progress              1.2.3      2023-12-06 [1] CRAN (R 4.3.2)
##  purrr               * 1.0.2      2023-08-10 [1] CRAN (R 4.3.2)
##  R6                    2.5.1      2021-08-19 [1] CRAN (R 4.3.2)
##  rappdirs              0.3.3      2021-01-31 [1] CRAN (R 4.3.2)
##  RColorBrewer          1.1-3      2022-04-03 [1] CRAN (R 4.3.2)
##  Rcpp                  1.0.12     2024-01-09 [1] CRAN (R 4.3.2)
##  RCurl                 1.98-1.14  2024-01-09 [1] CRAN (R 4.3.2)
##  readr               * 2.1.5      2024-01-10 [1] CRAN (R 4.3.2)
```

```
##  restfulr               0.0.15      2022-06-16 [1] CRAN (R 4.3.2)
##  rjson                  0.2.21      2022-01-09 [1] CRAN (R 4.3.2)
##  rlang                  1.1.3       2024-01-10 [1] CRAN (R 4.3.2)
##  rmarkdown              2.25        2023-09-18 [1] CRAN (R 4.3.2)
##  rprojroot              2.0.4       2023-11-05 [1] CRAN (R 4.3.2)
##  Rsamtools              2.18.0      2023-10-24 [1] Bioconductor
##  RSQLite                2.3.5       2024-01-21 [1] CRAN (R 4.3.2)
##  rstudioapi             0.15.0      2023-07-07 [1] CRAN (R 4.3.2)
##  rtracklayer            1.62.0      2023-10-24 [1] Bioconductor
##  S4Arrays               1.2.0       2023-10-24 [1] Bioconductor
##  S4Vectors              0.40.2      2023-11-23 [1] Bioconductor 3.18 (R 4.3.2)
##  scales                 1.3.0       2023-11-28 [1] CRAN (R 4.3.2)
##  seqinr                 4.2-36      2023-12-08 [1] CRAN (R 4.3.2)
##  sessioninfo            1.2.2       2021-12-06 [1] CRAN (R 4.3.2)
##  SparseArray            1.2.4       2024-02-11 [1] Bioconductor 3.18 (R 4.3.2)
##  statnet.common         4.9.0       2023-05-24 [1] CRAN (R 4.3.2)
##  stringi                1.8.3       2023-12-11 [1] CRAN (R 4.3.2)
##  stringr              * 1.5.1       2023-11-14 [1] CRAN (R 4.3.2)
##  SummarizedExperiment   1.32.0      2023-10-24 [1] Bioconductor
##  syntenet             * 1.4.0       2023-10-24 [1] Bioconductor
##  tibble               * 3.2.1       2023-03-20 [1] CRAN (R 4.3.2)
##  tidyr                * 1.3.1       2024-01-24 [1] CRAN (R 4.3.2)
##  tidyselect             1.2.0       2022-10-10 [1] CRAN (R 4.3.2)
##  tidyverse            * 2.0.0       2023-02-22 [1] CRAN (R 4.3.2)
##  timechange             0.3.0       2024-01-18 [1] CRAN (R 4.3.2)
##  tzdb                   0.4.0       2023-05-12 [1] CRAN (R 4.3.2)
##  utf8                   1.2.4       2023-10-22 [1] CRAN (R 4.3.2)
##  vctrs                  0.6.5       2023-12-01 [1] CRAN (R 4.3.2)
##  withr                  3.0.0       2024-01-16 [1] CRAN (R 4.3.2)
##  xfun                   0.42        2024-02-08 [1] CRAN (R 4.3.2)
##  XML                    3.99-0.16.1 2024-01-22 [1] CRAN (R 4.3.2)
##  xml2                   1.3.6       2023-12-04 [1] CRAN (R 4.3.2)
##  XVector                0.42.0      2023-10-24 [1] Bioconductor
##  yaml                   2.3.8       2023-12-11 [1] CRAN (R 4.3.2)
##  zlibbioc               1.48.0      2023-10-24 [1] Bioconductor
##
##  [1] /home/faalm/R/x86_64-pc-linux-gnu-library/4.3
##  [2] /usr/local/lib/R/site-library
##  [3] /usr/lib/R/site-library
##  [4] /usr/lib/R/library
##
## ------------------------------------------------------------------------------
```

Supplementary Text S4: Visual exploration of duplicated genes across the Eukarya tree of life

***Fabricio Almeida-Silva*[1] *and Yves Van de Peer*[1]**

[1]VIB-UGent Center for Plant Systems Biology, Ghent University, Ghent, Belgium

27 February 2024

Contents

1 Introduction 2
2 Loading data 3
3 Visualizing the frequency of duplicated genes by mode 3
4 BUSCO scores 11
5 Visualizing substitution rates for selected plant species 12
Saving objects 14
Session info 16

1 Introduction

Here, we will describe the code to perform exploratory data analyses on the duplicated gene frequencies in genomes from Ensembl instances.

To start, let's load the required data and packages.

```
set.seed(123) # for reproducibility

# Load required packages
library(doubletrouble)
## Registered S3 method overwritten by 'ggnetwork':
##   method         from
##   fortify.igraph ggtree
library(here)
## here() starts at /home/faalm/Dropbox/package_benchmarks/doubletrouble_paper
library(ggtree)
## ggtree v3.10.0 For help: https://yulab-smu.top/treedata-book/
##
## If you use the ggtree package suite in published research, please cite
## the appropriate paper(s):
##
## Guangchuang Yu, David Smith, Huachen Zhu, Yi Guan, Tommy Tsan-Yuk Lam.
## ggtree: an R package for visualization and annotation of phylogenetic
## trees with their covariates and other associated data. Methods in
## Ecology and Evolution. 2017, 8(1):28-36. doi:10.1111/2041-210X.12628
##
## G Yu. Data Integration, Manipulation and Visualization of Phylogenetic
## Trees (1st ed.). Chapman and Hall/CRC. 2022. ISBN: 9781032233574
##
## Shuangbin Xu, Lin Li, Xiao Luo, Meijun Chen, Wenli Tang, Li Zhan, Zehan
## Dai, Tommy T. Lam, Yi Guan, Guangchuang Yu. Ggtree: A serialized data
## object for visualization of a phylogenetic tree and annotation data.
## iMeta 2022, 1(4):e56. doi:10.1002/imt2.56
library(tidyverse)
## -- Attaching core tidyverse packages ------------------------ tidyverse 2.0.0 --
## v dplyr     1.1.4     v readr     2.1.5
## v forcats   1.0.0     v stringr   1.5.1
## v ggplot2   3.4.4     v tibble    3.2.1
## v lubridate 1.9.3     v tidyr     1.3.1
## v purrr     1.0.2
## -- Conflicts ------------------------------------------ tidyverse_conflicts() --
## x tidyr::expand() masks ggtree::expand()
## x dplyr::filter() masks stats::filter()
## x dplyr::lag()    masks stats::lag()
## i Use the conflicted package (<http://conflicted.r-lib.org/>) to force all conflicts to become errors
library(patchwork)

source(here("code", "utils.R"))
source(here("code", "utils_visualization.R"))
```

2 Loading data

First, we will load objects with species trees, duplicates per species, and BUSCO scores.

```
# Load metadata
load(here("products", "result_files", "metadata_all.rda"))

# Load BUSCO scores
load(here("products", "result_files", "busco_scores", "fungi_busco_scores.rda"))
load(here("products", "result_files", "busco_scores", "protists_busco_scores.rda"))
load(here("products", "result_files", "busco_scores", "plants_busco_scores.rda"))
load(here("products", "result_files", "busco_scores", "metazoa_busco_scores.rda"))
load(here("products", "result_files", "busco_scores", "vertebrates_busco_scores.rda"))

# Load trees
load(here("products", "result_files", "trees", "fungi_busco_trees.rda"))
load(here("products", "result_files", "trees", "protists_busco_trees.rda"))
load(here("products", "result_files", "trees", "plants_busco_trees.rda"))
load(here("products", "result_files", "trees", "metazoa_busco_trees.rda"))
load(here("products", "result_files", "trees", "vertebrates_busco_trees.rda"))

# Load duplicated genes
load(here("products", "result_files", "fungi_duplicates_unique.rda"))
load(here("products", "result_files", "protists_duplicates_unique.rda"))
load(here("products", "result_files", "plants_duplicates_unique.rda"))
load(here("products", "result_files", "vertebrates_duplicates_unique.rda"))
load(here("products", "result_files", "metazoa_duplicates_unique.rda"))

# Load substitution rates for plants
load(here("products", "result_files", "plants_kaks.rda"))
```

3 Visualizing the frequency of duplicated genes by mode

Now, we will visualize the frequency of duplicated genes by mode for each species. For that, we will first convert the list of duplicates into a long-formatted data frame, and clean tip labels in our species trees.

```
# Rename tip labels of trees
tree_fungi <- fungi_busco_trees$conc
tree_fungi$tip.label <- gsub("\\.", "_", tree_fungi$tip.label)

tree_protists <- protists_busco_trees$conc
tree_protists$tip.label <- gsub("\\.", "_", tree_protists$tip.label)

tree_plants <- plants_busco_trees$conc
tree_plants$tip.label <- gsub("\\.", "_", tree_plants$tip.label)

tree_metazoa <- metazoa_busco_trees$conc
```

```r
tree_metazoa$tip.label <- gsub("\\.", "_", tree_metazoa$tip.label)

tree_vertebrates <- vertebrates_busco_trees$conc
tree_vertebrates$tip.label <- gsub("\\.", "_", tree_vertebrates$tip.label)

# Get count tables
counts_fungi <- duplicates2counts(fungi_duplicates_unique)
counts_protists <- duplicates2counts(protists_duplicates_unique)
counts_plants <- duplicates2counts(plants_duplicates_unique)
counts_vertebrates <- duplicates2counts(vertebrates_duplicates_unique)
counts_metazoa <- duplicates2counts(metazoa_duplicates_unique)
```

Now, we will plot the trees with data for each Ensembl instance.

```r
# Fungi
p_fungi_tree <- plot_tree_taxa(
    tree = tree_fungi,
    metadata = metadata_all$fungi,
    taxon = "phylum",
    text_size = 2.5
)

p_fungi <- wrap_plots(
    # Plot 1: Species tree
    p_fungi_tree,
    # Plot 2: Duplicate relative frequency by mode
    plot_duplicate_freqs(
        counts_fungi |>
            mutate(
                species = factor(species, levels = rev(get_taxa_name(p_fungi_tree)))
            ),
        plot_type = "stack_percent"
    ) +
        theme(
            axis.text.y = element_blank(),
            axis.ticks.y = element_blank()
        ) +
        labs(y = NULL),
    widths = c(1, 4)
) +
    plot_annotation(title = "Fungi") &
    theme(plot.margin = margin(2, 0, 0, 2))

# Protists
p_protists_tree <- plot_tree_taxa(
    tree = tree_protists,
    metadata = metadata_all$protists |>
        filter(phylum != "Evosea"),
    taxon = "phylum",
    min_n_lab = 2,
    padding_text = 0.2,
```

```
        text_size = 2.5
    )
    p_protists <- wrap_plots(
        # Plot 1: Species tree
        p_protists_tree,
        # Plot 2: Duplicate relative frequency by mode
        plot_duplicate_freqs(
            counts_protists |>
                mutate(
                    species = factor(species, levels = rev(get_taxa_name(p_protists_tree)))
                ),
            plot_type = "stack_percent") +
            theme(
                axis.text.y = element_blank(),
                axis.ticks.y = element_blank()
            ) +
            labs(y = NULL),
        widths = c(1, 4)
    ) +
        plot_annotation(title = "Protists") &
        theme(plot.margin = margin(2, 0, 0, 2))


    # Plants
    p_plants_tree <- plot_tree_taxa(
        tree = tree_plants,
        metadata = metadata_all$plants,
        taxon = "order",
        min_n_lab = 3,
        text_size = 2.5
    )
    p_plants <- wrap_plots(
        # Plot 1: Species tree
        p_plants_tree,
        # Plot 2: Duplicate relative frequency by mode
        plot_duplicate_freqs(
            counts_plants |>
                mutate(
                    species = factor(species, levels = rev(get_taxa_name(p_plants_tree)))
                ),
            plot_type = "stack_percent") +
            theme(
                axis.text.y = element_blank(),
                axis.ticks.y = element_blank()
            ) +
            labs(y = NULL),
        widths = c(1, 4)
    ) +
        plot_annotation(title = "Plants") &
        theme(plot.margin = margin(2, 0, 0, 2))
```

```r
# Metazoa
p_metazoa_tree <- plot_tree_taxa(
    tree = tree_metazoa,
    metadata = metadata_all$metazoa |>
        filter(class != "Myxozoa"),
    taxon = "phylum",
    min_n = 2,
    text_size = 2.2,
    padding_text = 2
)
p_metazoa <- wrap_plots(
    # Plot 1: Species tree
    p_metazoa_tree,
    # Plot 2: Duplicate relative frequency by mode
    plot_duplicate_freqs(
        counts_metazoa |>
            mutate(
                species = factor(species, levels = rev(get_taxa_name(p_metazoa_tree)))
            ),
        plot_type = "stack_percent") +
        theme(
            axis.text.y = element_blank(),
            axis.ticks.y = element_blank()
        ) +
        labs(y = NULL),
    widths = c(1, 4)
) +
    plot_annotation(title = "Metazoa") &
    theme(plot.margin = margin(2, 0, 0, 2))

# Vertebrates
p_vertebrates_tree <- plot_tree_taxa(
    tree = tree_vertebrates,
    metadata = metadata_all$ensembl |>
        mutate(class = replace_na(class, "Other")),
    taxon = "class",
    min_n = 2,
    text_size = 2.5
)
p_vertebrates <- wrap_plots(
    # Plot 1: Species tree
    p_vertebrates_tree,
    # Plot 2: Duplicate relative frequency by mode
    plot_duplicate_freqs(
        counts_vertebrates |>
            mutate(
                species = factor(species, levels = rev(get_taxa_name(p_vertebrates_tree)))
            ),
        plot_type = "stack_percent") +
        theme(
            axis.text.y = element_blank(),
```

```
                axis.ticks.y = element_blank()
            ) +
            labs(y = NULL),
        widths = c(1, 4)
    ) +
        plot_annotation(title = "Vertebrates") &
        theme(plot.margin = margin(2, 0, 0, 2))

    # Combining all figures into one
    p_duplicates_all_ensembl <- wrap_plots(
        wrap_plots(
            p_protists +
                theme(legend.position = "none") +
                labs(title = "Protists", x = NULL),
            p_fungi +
                theme(legend.position = "none") +
                ggtitle("Fungi"),
            nrow = 2, heights = c(1, 2)
        ),
        p_plants + theme(legend.position = "none") + ggtitle("Plants"),
        p_metazoa + theme(legend.position = "none") + ggtitle("Metazoa (Invertebrates)"),
        p_vertebrates + ggtitle("Ensembl (Vertebrates)"),
        nrow = 1
    ) +
        plot_layout(axis_titles = "collect")
```

```
    p_duplicates_all_ensembl
```

Supplementary Text S4: Visual exploration of duplicated genes across the Eukarya tree of life

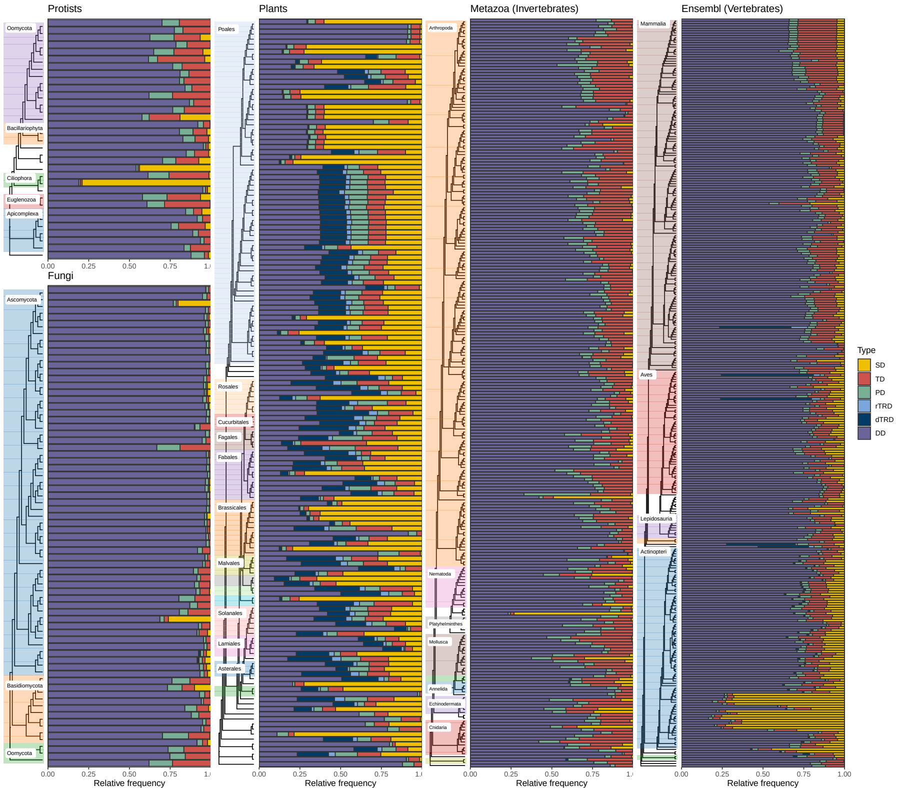

By visually comparing the Ensembl instances, we can see that plant genomes have a much greater abundance of segmental duplicates, possibly due to pervasive whole-genome duplication events. However, other major branches of the Eukarya tree of life also have particular species with a high proportion of SD-derived genes. Notably, while SD events are widespread in plants, vertebrate species with high proportions of SD-derived genes are concentrated in a particular branch (teleost fishes). To investigate that, we will highlight species for which at least 20% of the duplicated genes derived from segmental duplications.

```
# For each Ensembl instance, show species with >=20% of genes derived from SD
## Define helper function
sd_abundant <- function(count_table, min = 20) {

    perc_table <- count_table |>
        group_by(species) |>
        mutate(percentage = (n / sum(n)) * 100) |>
        ungroup() |>
        filter(type == "SD", percentage >= min)

    return(perc_table)
}

# Get a table of SD-abundant species for each instance
sd_abundant_spp <- bind_rows(
    sd_abundant(counts_fungi) |> mutate(instance = "fungi"),
    sd_abundant(counts_protists) |> mutate(instance = "protists"),
    sd_abundant(counts_plants) |> mutate(instance = "plants"),
```

```
    sd_abundant(counts_vertebrates) |> mutate(instance = "vertebrates"),
    sd_abundant(counts_metazoa) |> mutate(instance = "metazoa")
) |>
    as.data.frame()
```

Then, let's summarize the frequencies (absolute and relative) in a table.

```
# How many species per instance?
sd_abundant_spp |>
    count(instance) |>
    mutate(
        percentage = n / c(
            nrow(metadata_all$fungi),
            nrow(metadata_all$metazoa),
            nrow(metadata_all$plants),
            nrow(metadata_all$protists),
            nrow(metadata_all$ensembl)
        ) * 100
    )
##      instance  n percentage
## 1       fungi  2   2.857143
## 2     metazoa  7   2.766798
## 3      plants 94  63.087248
## 4    protists  2   6.060606
## 5 vertebrates 21   6.624606
```

Once again, our findings highlight the abundance of large-scale duplications in plant genomes, as segmental duplications contributed to 20% of the duplicated genes in 94 species (63%). Next, let's print all SD-abundant species.

```
# Show all species
knitr::kable(sd_abundant_spp)
```

type	n	species	percentage	instance
SD	2138	fusarium_oxysporum	20.16030	fungi
SD	683	saccharomyces_cerevisiae	26.16858	fungi
SD	14351	emiliania_huxleyi	43.78509	protists
SD	27661	paramecium_tetraurelia	79.03369	protists
SD	22430	actinidia_chinensis	72.54908	plants
SD	4357	ananas_comosus	22.15048	plants
SD	5885	arabidopsis_halleri	21.37202	plants
SD	7530	arabidopsis_thaliana	33.29796	plants
SD	42997	avena_sativa_ot3098	74.43306	plants
SD	63492	avena_sativa_sang	79.22833	plants
SD	7461	brachypodium_distachyon	27.24285	plants
SD	48381	brassica_juncea	69.66407	plants
SD	59918	brassica_napus	63.31417	plants
SD	24314	brassica_oleracea	44.30232	plants
SD	23541	brassica_rapa	62.58907	plants
SD	19282	brassica_rapa_ro18	49.29567	plants
SD	69355	camelina_sativa	79.45628	plants
SD	15785	chenopodium_quinoa	48.57371	plants
SD	3600	citrullus_lanatus	22.76608	plants
SD	4084	coffea_canephora	20.60545	plants
SD	3317	cynara_cardunculus	20.20959	plants
SD	41246	digitaria_exilis	75.51999	plants
SD	8809	dioscorea_rotundata	32.30764	plants
SD	66262	echinochloa_crusgalli	70.97016	plants
SD	13447	eragrostis_curvula	27.40650	plants
SD	46488	eucalyptus_grandis	75.45773	plants
SD	6846	ficus_carica	30.61580	plants
SD	6747	galdieria_sulphuraria	25.40573	plants
SD	36994	glycine_max	72.19468	plants
SD	16290	gossypium_raimondii	48.65882	plants
SD	13432	helianthus_annuus	23.21425	plants
SD	9537	ipomoea_triloba	35.65100	plants
SD	13096	juglans_regia	34.16556	plants
SD	7374	kalanchoe_fedtschenkoi	28.37900	plants
SD	6757	lactuca_sativa	20.52178	plants
SD	5758	leersia_perrieri	25.97321	plants
SD	16167	lupinus_angustifolius	54.14448	plants
SD	22987	malus_domestica_golden	62.58031	plants
SD	14278	manihot_esculenta	52.81693	plants
SD	16170	musa_acuminata	54.10017	plants
SD	4586	nymphaea_colorata	20.98472	plants
SD	5092	oryza_barthii	20.55464	plants
SD	5078	oryza_brachyantha	23.83366	plants
SD	6054	oryza_glaberrima	23.77567	plants
SD	5454	oryza_glumipatula	21.46484	plants
SD	5847	oryza_punctata	24.25437	plants
SD	5587	oryza_rufipogon	21.24335	plants
SD	6096	oryza_sativa	23.94344	plants
SD	6136	oryza_sativa_arc	22.25769	plants
SD	6139	oryza_sativa_azucena	22.19370	plants
SD	6186	oryza_sativa_chaomeo	22.05583	plants
SD	6125	oryza_sativa_gobolsailbalam	22.38506	plants
SD	6207	oryza_sativa_ir64	22.73876	plants
SD	6131	oryza_sativa_ketannangka	22.16238	plants
SD	6479	oryza_sativa_khaoyaiguang	23.36964	plants
SD	6144	oryza_sativa_larhamugad	22.46107	plants

4 BUSCO scores

Next, we will test whether the percentage of segmental duplicates in genomes is associated with the percentage of complete BUSCOs. In other words, we want to find out whether the low percentages of SD gene pairs is due to genome fragmentation.

```
# Define function to plot association between % SD and % complete BUSCOs
plot_busco_sd_assoc <- function(busco_df, counts_table) {

    p <- busco_df |>
        filter(Class %in% c("Complete_SC", "Complete_duplicate")) |>
        mutate(species = str_replace_all(File, "\\.fa", "")) |>
        mutate(species = str_replace_all(species, "\\.", "_")) |>
        group_by(species) |>
        summarise(complete_BUSCOs = sum(Frequency)) |>
        inner_join(sd_abundant(counts_table, min = 0)) |>
        ggpubr::ggscatter(
            x = "complete_BUSCOs", y = "percentage",
            color = "deepskyblue4", alpha = 0.4,
            add = "reg.line", add.params = list(
                color = "black", fill = "lightgray"
            ),
            conf.int = TRUE,
            cor.coef = TRUE,
            cor.coeff.args = list(
                method = "pearson", label.x = 3, label.sep = "\n"
            )
        ) +
        labs(x = "% complete BUSCOs", y = "% SD duplicates")

    return(p)
}

# Fungi
p_busco_association <- patchwork::wrap_plots(
    plot_busco_sd_assoc(fungi_busco_scores, counts_fungi) +
        labs(title = "Fungi"),
    plot_busco_sd_assoc(plants_busco_scores, counts_plants) +
        labs(title = "Plants"),
    plot_busco_sd_assoc(protists_busco_scores, counts_protists) +
        labs(title = "Protists"),
    plot_busco_sd_assoc(metazoa_busco_scores, counts_metazoa) +
        labs(title = "Metazoa"),
    plot_busco_sd_assoc(vertebrates_busco_scores, counts_vertebrates) +
        labs(title = "Vertebrates"),
    nrow = 1
)
```

```
p_busco_association
```

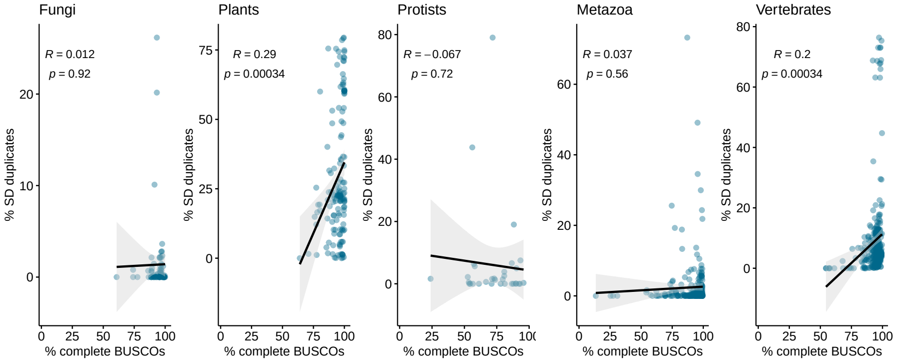

There is weak or no association between the percentage of complete BUSCOs and the percentage of SD-derived genes.

5 Visualizing substitution rates for selected plant species

Here, we will first visualize K_s distributions for *Glycine max* and *Phaseolus vulgaris* by mode of duplication.

```
# G. max
gmax_ks_distro <- plot_ks_distro(
    plants_kaks$glycine_max, max_ks = 2, bytype = TRUE, binwidth = 0.03
) +
    labs(title = NULL, y = NULL)

# P. vulgaris
pvu_ks_distro <- plot_ks_distro(
    plants_kaks$phaseolus_vulgaris, max_ks = 2, bytype = TRUE, binwidth = 0.03
) +
    labs(title = NULL, y = NULL)


# Combining plots
p_ks_legumes <- wrap_plots(
    gmax_ks_distro +
        labs(title = "Glycine max") +
        theme(plot.title = element_text(face = "italic")),
    pvu_ks_distro +
        labs(title = "Phaseolus vulgaris") +
        theme(plot.title = element_text(face = "italic")),
    nrow = 1
)
```

```
p_ks_legumes
```

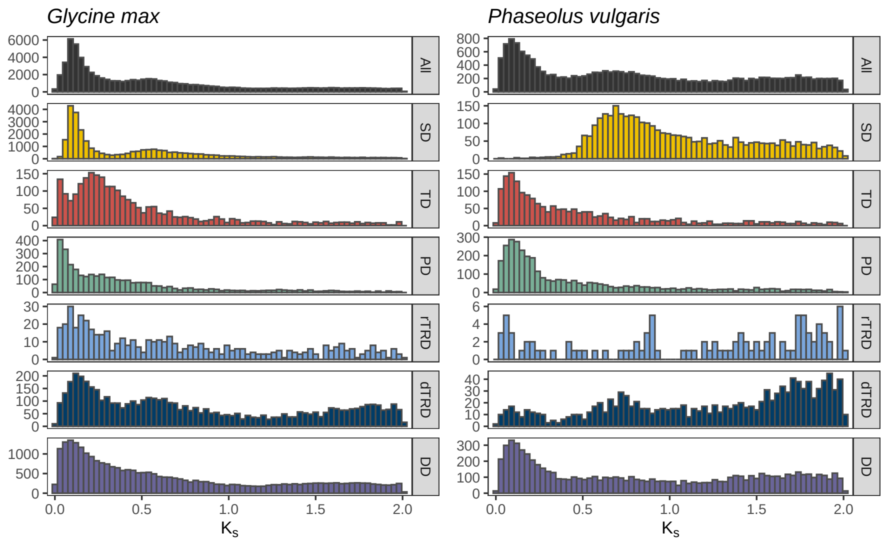

The plot shows the importance of visualizing Ks distributions by mode. When visualizing the whole-paranome distribution, detection of peaks is not trivial, and potential whole-genome duplication events might be masked. When we split the distribution by mode of duplication, we can more easily observe segmental duplicates that cluster together, providing strong evidence for whole-genome duplication events (2 events for *G. max*, and 1 events for *P. vulgaris*).

Next, we will plot the distributions of K_a, K_s, and K_a/K_s values for selected plant species with phylogenetic context.

```
# Subset plant tree to get selected species only
tree_subset <- ape::keep.tip(tree_plants, names(plants_kaks))

# Clean names
names(plants_kaks) <- gsub("_", " ", str_to_title(names(plants_kaks)))
tree_subset$tip.label <- gsub("_", " ", str_to_title(tree_subset$tip.label))

# Plot tree
p_tree_selected <- ggtree(tree_subset, branch.length = "none") +
    geom_tiplab(fontface = "italic", size = 3)

# Reoder rates list based on tree topology
ord <- rev(ggtree::get_taxa_name(p_tree_selected))
rl <- plants_kaks[ord]

# Plot rates by species with tree on the left
p_rates_phylogeny <- wrap_plots(
    p_tree_selected + xlim(0, 10),
    plot_rates_by_species(rl, rate_column = "Ks", range = c(0, 2)) +
        theme(
            axis.text.y = element_blank(),
            axis.ticks.y = element_blank()
```

```
        ),
    plot_rates_by_species(
        rl, rate_column = "Ka", range = c(0, 2),
        fill = "mediumseagreen", color = "seagreen"
    ) +
        theme(
            axis.text.y = element_blank(),
            axis.ticks.y = element_blank()
        ),
    plot_rates_by_species(
        rl, rate_column = "Ka_Ks", range = c(0, 2),
        fill = "darkorange2", color = "darkorange3"
    ) +
        theme(
            axis.text.y = element_blank(),
            axis.ticks.y = element_blank()
        ),
    nrow = 1
) +
    plot_annotation(title = "Substitution rates in a phylogenetic context")
```

```
p_rates_phylogeny
```

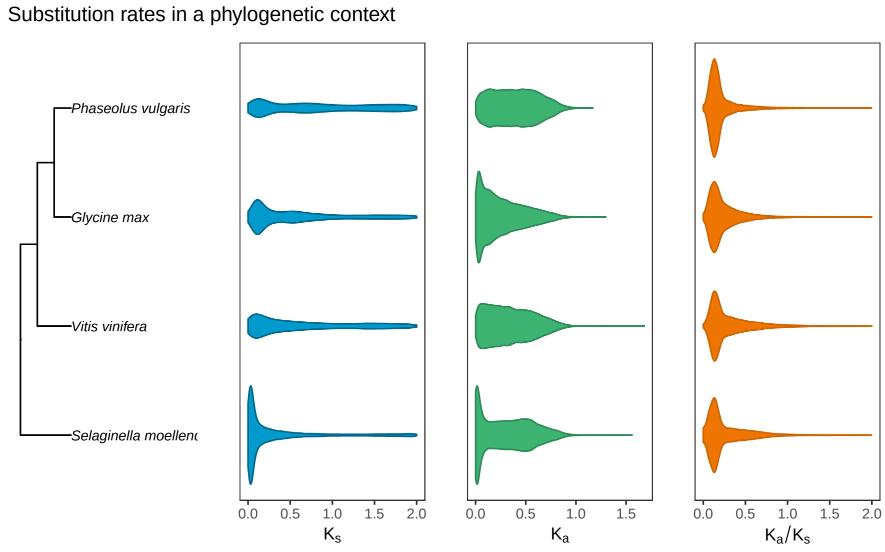

Saving objects

Finally, let's save important objects created in this session for further use.

```
# Save plots for each instance
save(
    p_fungi, compress = "xz",
    file = here("products", "plots", "p_fungi.rda")
```

```
        )

        save(
            p_metazoa, compress = "xz",
            file = here("products", "plots", "p_metazoa.rda")
        )

        save(
            p_protists, compress = "xz",
            file = here("products", "plots", "p_protists.rda")
        )

        save(
            p_vertebrates, compress = "xz",
            file = here("products", "plots", "p_vertebrates.rda")
        )

        save(
            p_plants, compress = "xz",
            file = here("products", "plots", "p_plants.rda")
        )

        save(
            p_duplicates_all_ensembl, compress = "xz",
            file = here("products", "plots", "p_duplicates_all_ensembl.rda")
        )

        save(
            p_ks_legumes, compress = "xz",
            file = here("products", "plots", "p_ks_legumes.rda")
        )

        save(
            p_rates_phylogeny, compress = "xz",
            file = here("products", "plots", "p_rates_phylogeny.rda")
        )

        save(
            p_busco_association, compress = "xz",
            file = here("products", "plots", "p_busco_association.rda")
        )

        # Save tables
        save(
            sd_abundant_spp, compress = "xz",
            file = here("products", "result_files", "sd_abundant_spp.rda")
        )
```

Session info

This document was created under the following conditions:

```
## - Session info ---------------------------------------------------------------
##  setting  value
##  version  R version 4.3.2 (2023-10-31)
##  os       Ubuntu 22.04.3 LTS
##  system   x86_64, linux-gnu
##  ui       X11
##  language (EN)
##  collate  en_US.UTF-8
##  ctype    en_US.UTF-8
##  tz       Europe/Brussels
##  date     2024-02-27
##  pandoc   3.1.1 @ /usr/lib/rstudio/resources/app/bin/quarto/bin/tools/ (via rmarkdown)
##
## - Packages -------------------------------------------------------------------
##  package              * version     date (UTC) lib source
##  abind                  1.4-5       2016-07-21 [1] CRAN (R 4.3.2)
##  ade4                   1.7-22      2023-02-06 [1] CRAN (R 4.3.2)
##  AnnotationDbi          1.64.1      2023-11-03 [1] Bioconductor
##  ape                    5.7-1       2023-03-13 [1] CRAN (R 4.3.2)
##  aplot                  0.2.2       2023-10-06 [1] CRAN (R 4.3.2)
##  backports              1.4.1       2021-12-13 [1] CRAN (R 4.3.2)
##  Biobase                2.62.0      2023-10-24 [1] Bioconductor
##  BiocFileCache          2.10.1      2023-10-26 [1] Bioconductor
##  BiocGenerics           0.48.1      2023-11-01 [1] Bioconductor
##  BiocIO                 1.12.0      2023-10-24 [1] Bioconductor
##  BiocManager            1.30.22     2023-08-08 [1] CRAN (R 4.3.2)
##  BiocParallel           1.37.0      2024-01-19 [1] Github (Bioconductor/BiocParallel@79a1b2d)
##  BiocStyle            * 2.30.0      2023-10-24 [1] Bioconductor
##  biomaRt                2.58.2      2024-01-30 [1] Bioconductor 3.18 (R 4.3.2)
##  Biostrings             2.70.2      2024-01-28 [1] Bioconductor 3.18 (R 4.3.2)
##  bit                    4.0.5       2022-11-15 [1] CRAN (R 4.3.2)
##  bit64                  4.0.5       2020-08-30 [1] CRAN (R 4.3.2)
##  bitops                 1.0-7       2021-04-24 [1] CRAN (R 4.3.2)
##  blob                   1.2.4       2023-03-17 [1] CRAN (R 4.3.2)
##  bookdown               0.37        2023-12-01 [1] CRAN (R 4.3.2)
##  broom                  1.0.5       2023-06-09 [1] CRAN (R 4.3.2)
##  cachem                 1.0.8       2023-05-01 [1] CRAN (R 4.3.2)
##  car                    3.1-2       2023-03-30 [1] CRAN (R 4.3.2)
##  carData                3.0-5       2022-01-06 [1] CRAN (R 4.3.2)
##  cli                    3.6.2       2023-12-11 [1] CRAN (R 4.3.2)
##  coda                   0.19-4.1    2024-01-31 [1] CRAN (R 4.3.2)
##  codetools              0.2-19      2023-02-01 [4] CRAN (R 4.2.2)
##  colorspace             2.1-0       2023-01-23 [1] CRAN (R 4.3.2)
##  crayon                 1.5.2       2022-09-29 [1] CRAN (R 4.3.2)
##  curl                   5.2.0       2023-12-08 [1] CRAN (R 4.3.2)
##  DBI                    1.2.1       2024-01-12 [1] CRAN (R 4.3.2)
##  dbplyr                 2.4.0       2023-10-26 [1] CRAN (R 4.3.2)
```

```
##  DelayedArray           0.28.0     2023-10-24 [1] Bioconductor
##  digest                 0.6.34     2024-01-11 [1] CRAN (R 4.3.2)
##  doParallel             1.0.17     2022-02-07 [1] CRAN (R 4.3.2)
##  doubletrouble        * 1.3.4      2024-02-05 [1] Bioconductor
##  dplyr                * 1.1.4      2023-11-17 [1] CRAN (R 4.3.2)
##  evaluate               0.23       2023-11-01 [1] CRAN (R 4.3.2)
##  fansi                  1.0.6      2023-12-08 [1] CRAN (R 4.3.2)
##  farver                 2.1.1      2022-07-06 [1] CRAN (R 4.3.2)
##  fastmap                1.1.1      2023-02-24 [1] CRAN (R 4.3.2)
##  filelock               1.0.3      2023-12-11 [1] CRAN (R 4.3.2)
##  forcats              * 1.0.0      2023-01-29 [1] CRAN (R 4.3.2)
##  foreach                1.5.2      2022-02-02 [1] CRAN (R 4.3.2)
##  fs                     1.6.3      2023-07-20 [1] CRAN (R 4.3.2)
##  generics               0.1.3      2022-07-05 [1] CRAN (R 4.3.2)
##  GenomeInfoDb           1.38.6     2024-02-08 [1] Bioconductor 3.18 (R 4.3.2)
##  GenomeInfoDbData       1.2.11     2023-12-21 [1] Bioconductor
##  GenomicAlignments      1.38.2     2024-01-16 [1] Bioconductor 3.18 (R 4.3.2)
##  GenomicFeatures        1.54.3     2024-01-31 [1] Bioconductor 3.18 (R 4.3.2)
##  GenomicRanges          1.54.1     2023-10-29 [1] Bioconductor
##  ggfun                  0.1.4      2024-01-19 [1] CRAN (R 4.3.2)
##  ggnetwork              0.5.13     2024-02-14 [1] CRAN (R 4.3.2)
##  ggplot2              * 3.4.4      2023-10-12 [1] CRAN (R 4.3.2)
##  ggplotify              0.1.2      2023-08-09 [1] CRAN (R 4.3.2)
##  ggpubr                 0.6.0.999  2024-02-09 [1] Github (kassambara/ggpubr@6aeb4f7)
##  ggsignif               0.6.4      2022-10-13 [1] CRAN (R 4.3.2)
##  ggtree               * 3.10.0     2023-10-24 [1] Bioconductor
##  glue                   1.7.0      2024-01-09 [1] CRAN (R 4.3.2)
##  gridGraphics           0.5-1      2020-12-13 [1] CRAN (R 4.3.2)
##  gtable                 0.3.4      2023-08-21 [1] CRAN (R 4.3.2)
##  here                 * 1.0.1      2020-12-13 [1] CRAN (R 4.3.2)
##  hms                    1.1.3      2023-03-21 [1] CRAN (R 4.3.2)
##  htmltools              0.5.7      2023-11-03 [1] CRAN (R 4.3.2)
##  htmlwidgets            1.6.4      2023-12-06 [1] CRAN (R 4.3.2)
##  httr                   1.4.7      2023-08-15 [1] CRAN (R 4.3.2)
##  igraph                 2.0.1.1    2024-01-30 [1] CRAN (R 4.3.2)
##  intergraph             2.0-4      2024-02-01 [1] CRAN (R 4.3.2)
##  IRanges                2.36.0     2023-10-24 [1] Bioconductor
##  iterators              1.0.14     2022-02-05 [1] CRAN (R 4.3.2)
##  jsonlite               1.8.8      2023-12-04 [1] CRAN (R 4.3.2)
##  KEGGREST               1.42.0     2023-10-24 [1] Bioconductor
##  knitr                  1.45       2023-10-30 [1] CRAN (R 4.3.2)
##  labeling               0.4.3      2023-08-29 [1] CRAN (R 4.3.2)
##  lattice                0.22-5     2023-10-24 [4] CRAN (R 4.3.1)
##  lazyeval               0.2.2      2019-03-15 [1] CRAN (R 4.3.2)
##  lifecycle              1.0.4      2023-11-07 [1] CRAN (R 4.3.2)
##  lubridate            * 1.9.3      2023-09-27 [1] CRAN (R 4.3.2)
##  magrittr               2.0.3      2022-03-30 [1] CRAN (R 4.3.2)
##  MASS                   7.3-60     2023-05-04 [4] CRAN (R 4.3.1)
##  Matrix                 1.6-3      2023-11-14 [4] CRAN (R 4.3.2)
##  MatrixGenerics         1.14.0     2023-10-24 [1] Bioconductor
##  matrixStats            1.2.0      2023-12-11 [1] CRAN (R 4.3.2)
```

```
##  mclust                   6.0.1      2023-11-15 [1] CRAN (R 4.3.2)
##  memoise                  2.0.1      2021-11-26 [1] CRAN (R 4.3.2)
##  mgcv                     1.9-0      2023-07-11 [4] CRAN (R 4.3.1)
##  MSA2dist                 1.6.0      2023-10-24 [1] Bioconductor
##  munsell                  0.5.0      2018-06-12 [1] CRAN (R 4.3.2)
##  network                  1.18.2     2023-12-05 [1] CRAN (R 4.3.2)
##  networkD3                0.4        2017-03-18 [1] CRAN (R 4.3.2)
##  nlme                     3.1-163    2023-08-09 [4] CRAN (R 4.3.1)
##  patchwork              * 1.2.0      2024-01-08 [1] CRAN (R 4.3.2)
##  pheatmap                 1.0.12     2019-01-04 [1] CRAN (R 4.3.2)
##  pillar                   1.9.0      2023-03-22 [1] CRAN (R 4.3.2)
##  pkgconfig                2.0.3      2019-09-22 [1] CRAN (R 4.3.2)
##  png                      0.1-8      2022-11-29 [1] CRAN (R 4.3.2)
##  prettyunits              1.2.0      2023-09-24 [1] CRAN (R 4.3.2)
##  progress                 1.2.3      2023-12-06 [1] CRAN (R 4.3.2)
##  purrr                  * 1.0.2      2023-08-10 [1] CRAN (R 4.3.2)
##  R6                       2.5.1      2021-08-19 [1] CRAN (R 4.3.2)
##  rappdirs                 0.3.3      2021-01-31 [1] CRAN (R 4.3.2)
##  RColorBrewer             1.1-3      2022-04-03 [1] CRAN (R 4.3.2)
##  Rcpp                     1.0.12     2024-01-09 [1] CRAN (R 4.3.2)
##  RCurl                    1.98-1.14  2024-01-09 [1] CRAN (R 4.3.2)
##  readr                  * 2.1.5      2024-01-10 [1] CRAN (R 4.3.2)
##  restfulr                 0.0.15     2022-06-16 [1] CRAN (R 4.3.2)
##  rjson                    0.2.21     2022-01-09 [1] CRAN (R 4.3.2)
##  rlang                    1.1.3      2024-01-10 [1] CRAN (R 4.3.2)
##  rmarkdown                2.25       2023-09-18 [1] CRAN (R 4.3.2)
##  rprojroot                2.0.4      2023-11-05 [1] CRAN (R 4.3.2)
##  Rsamtools                2.18.0     2023-10-24 [1] Bioconductor
##  RSQLite                  2.3.5      2024-01-21 [1] CRAN (R 4.3.2)
##  rstatix                  0.7.2      2023-02-01 [1] CRAN (R 4.3.2)
##  rstudioapi               0.15.0     2023-07-07 [1] CRAN (R 4.3.2)
##  rtracklayer              1.62.0     2023-10-24 [1] Bioconductor
##  S4Arrays                 1.2.0      2023-10-24 [1] Bioconductor
##  S4Vectors                0.40.2     2023-11-23 [1] Bioconductor 3.18 (R 4.3.2)
##  scales                   1.3.0      2023-11-28 [1] CRAN (R 4.3.2)
##  seqinr                   4.2-36     2023-12-08 [1] CRAN (R 4.3.2)
##  sessioninfo              1.2.2      2021-12-06 [1] CRAN (R 4.3.2)
##  SparseArray              1.2.4      2024-02-11 [1] Bioconductor 3.18 (R 4.3.2)
##  statnet.common           4.9.0      2023-05-24 [1] CRAN (R 4.3.2)
##  stringi                  1.8.3      2023-12-11 [1] CRAN (R 4.3.2)
##  stringr                * 1.5.1      2023-11-14 [1] CRAN (R 4.3.2)
##  SummarizedExperiment     1.32.0     2023-10-24 [1] Bioconductor
##  syntenet                 1.4.0      2023-10-24 [1] Bioconductor
##  tibble                 * 3.2.1      2023-03-20 [1] CRAN (R 4.3.2)
##  tidyr                  * 1.3.1      2024-01-24 [1] CRAN (R 4.3.2)
##  tidyselect               1.2.0      2022-10-10 [1] CRAN (R 4.3.2)
##  tidytree                 0.4.6      2023-12-12 [1] CRAN (R 4.3.2)
##  tidyverse              * 2.0.0      2023-02-22 [1] CRAN (R 4.3.2)
##  timechange               0.3.0      2024-01-18 [1] CRAN (R 4.3.2)
##  treeio                   1.26.0     2023-10-24 [1] Bioconductor
##  tzdb                     0.4.0      2023-05-12 [1] CRAN (R 4.3.2)
```

```
##  utf8                   1.2.4      2023-10-22 [1] CRAN (R 4.3.2)
##  vctrs                  0.6.5      2023-12-01 [1] CRAN (R 4.3.2)
##  withr                  3.0.0      2024-01-16 [1] CRAN (R 4.3.2)
##  xfun                   0.42       2024-02-08 [1] CRAN (R 4.3.2)
##  XML                    3.99-0.16.1 2024-01-22 [1] CRAN (R 4.3.2)
##  xml2                   1.3.6      2023-12-04 [1] CRAN (R 4.3.2)
##  XVector                0.42.0     2023-10-24 [1] Bioconductor
##  yaml                   2.3.8      2023-12-11 [1] CRAN (R 4.3.2)
##  yulab.utils            0.1.4      2024-01-28 [1] CRAN (R 4.3.2)
##  zlibbioc               1.48.0     2023-10-24 [1] Bioconductor
##
##  [1] /home/faalm/R/x86_64-pc-linux-gnu-library/4.3
##  [2] /usr/local/lib/R/site-library
##  [3] /usr/lib/R/site-library
##  [4] /usr/lib/R/library
##
## ------------------------------------------------------------------------------
```

Supplementary Text S5: Runtime benchmark

Fabricio Almeida-Silva[1] ***and Yves Van de Peer***[1]

[1]VIB-UGent Center for Plant Systems Biology, Ghent University, Ghent, Belgium

26 July 2024

Contents

1 Introduction 2

2 Benchmark 1: `classify_gene_pairs()` 2

3 Benchmark 2: `pairs2kaks()` 4

4 Benchmark 3: **doubletrouble** vs **DupGen_finder** 5

Session info 10

1 Introduction

Here, we will perform a runtime benchmark for functions related to duplicate classification and substitution rates calculation using model organisms.

To start, let's load the required data and packages.

```
set.seed(123) # for reproducibility

# Load required packages
library(doubletrouble)
library(here)
## here() starts at /home/faalm/Dropbox/package_benchmarks/doubletrouble_paper
library(tidyverse)
## -- Attaching core tidyverse packages ------------------------ tidyverse 2.0.0 --
## v dplyr     1.1.4     v readr     2.1.5
## v forcats   1.0.0     v stringr   1.5.1
## v ggplot2   3.5.1     v tibble    3.2.1
## v lubridate 1.9.3     v tidyr     1.3.1
## v purrr     1.0.2
## -- Conflicts ------------------------------------------ tidyverse_conflicts() --
## x dplyr::filter() masks stats::filter()
## x dplyr::lag()    masks stats::lag()
## i Use the conflicted package (<http://conflicted.r-lib.org/>) to force all conflicts to become errors
library(patchwork)

source(here("code", "utils.R"))

# Load sample metadata for Ensembl instances
load(here("products", "result_files", "metadata_all.rda"))
```

2 Benchmark 1: `classify_gene_pairs()`

Here, we will benchmark the performance of `classify_gene_pairs()` with model organisms.

First, let's get the genome and annotation data.

```
# Create a data frame with names of model species and their Ensembl instances
model_species <- data.frame(
    species = c(
        "arabidopsis_thaliana", "caenorhabditis_elegans",
        "homo_sapiens", "saccharomyces_cerevisiae",
        "drosophila_melanogaster", "danio_rerio"
    ),
    instance = c(
        "plants", "metazoa", "ensembl", "fungi", "metazoa", "ensembl"
    )
)

# For each organism, download data, and identify and classify duplicates
model_duplicates <- lapply(seq_len(nrow(model_species)), function(x) {
```

```r
        species <- model_species$species[x]
        instance <- model_species$instance[x]

        # Get annotation
        annot <- get_annotation(model_species[x, ], instance)

        # Get proteome and keep only primary transcripts
        seq <- get_proteomes(model_species[x, ], instance)
        seq <- filter_sequences(seq, annot)

        # Process data
        pdata <- syntenet::process_input(seq, annot, filter_annotation = TRUE)

        # Perform DIAMOND search
        outdir <- file.path(tempdir(), paste0(species, "_intra"))
        diamond <- syntenet::run_diamond(
            seq = pdata$seq,
            compare = "intraspecies",
            outdir = outdir,
            threads = 4,
            ... = "--sensitive"
        )

        fs::dir_delete(outdir)

        # Classify duplicates - standard mode
        start <- Sys.time()
        duplicate_pairs <- classify_gene_pairs(
                blast_list = diamond,
                annotation = pdata$annotation,
                scheme = "standard"
            )[[1]]
        end <- Sys.time()
        runtime <- end - start

        return(runtime)
    })
    names(model_duplicates) <- gsub("_", " ", str_to_title(model_species$species))

    # Summarize results in a table
    benchmark_classification <- data.frame(
        species = names(model_duplicates),
        time_seconds = as.numeric(unlist(model_duplicates))
    )

    # Save results
    save(
        benchmark_classification, compress = "xz",
        file = here("products", "result_files", "benchmark_classification.rda")
    )
```

3 Benchmark 2: `pairs2kaks()`

Next, we will benchmark the performance of `pairs2kaks()` for duplicate pairs in the *Saccharomyces cerevisiae* genome. We will do it using a single thread, and using parallelization (with 4 and 8 threads).

First of all, let's get the required data for `pairs2kaks()`.

```
# Load duplicate pairs for S. cerevisiae
load(here("products", "result_files", "fungi_duplicates.rda"))
scerevisiae_pairs <- fungi_duplicates["saccharomyces_cerevisiae"]

# Get CDS for S. cerevisiae
scerevisiae_cds <- get_cds_ensembl("saccharomyces_cerevisiae", "fungi")
```

Now, we can do the benchmark.

```
# 1 thread
start <- Sys.time()
kaks <- pairs2kaks(
    scerevisiae_pairs,
    scerevisiae_cds,
    threads = 1
)
end <- Sys.time()
runtime1 <- end - start

# 4 threads
start <- Sys.time()
kaks <- pairs2kaks(
    scerevisiae_pairs,
    scerevisiae_cds,
    threads = 4
)
end <- Sys.time()
runtime4 <- end - start

# 8 threads
start <- Sys.time()
kaks <- pairs2kaks(
    scerevisiae_pairs,
    scerevisiae_cds,
    threads = 8
)
end <- Sys.time()
runtime8 <- end - start

# Summarize results in a table
benchmark_kaks <- data.frame(
    Threads = factor(c(1, 4, 8)),
    Time_minutes = as.numeric(c(runtime1, runtime4, runtime8))
) |>
```

```
    dplyr::mutate(
        Pairs_per_minute = nrow(scerevisiae_pairs[[1]]) / Time_minutes,
        Pairs_per_second = nrow(scerevisiae_pairs[[1]]) / (Time_minutes * 60)
    )
save(
    benchmark_kaks, compress = "xz",
    file = here("products", "result_files", "benchmark_kaks.rda")
)
```

4 Benchmark 3: **doubletrouble** vs **DupGen_finder**

Here, we will classify duplicate pairs in the *A. thaliana* genome using **doubletrouble** and DupGen_finder to assess if they produce the same results, and compare their runtimes.

First, let's get all data we need (proteomes, annotation, and DIAMOND tables).

```
# Get annotation
smeta <- data.frame(
    species = c("arabidopsis_thaliana", "amborella_trichopoda"),
    instance = "plants"
)
annot <- get_annotation(smeta, "plants")

# Get proteome and keep only primary transcripts
seq <- get_proteomes(smeta, "plants")
seq <- filter_sequences(seq, annot)

# Process data
pdata <- syntenet::process_input(seq, annot, filter_annotation = TRUE)

# Run intraspecies DIAMOND search
outdir <- file.path(tempdir(), "benchmark3_intra")
diamond_intra <- syntenet::run_diamond(
    seq = pdata$seq,
    compare = "intraspecies",
    outdir = outdir,
    threads = 4,
    ... = "--sensitive"
)
fs::dir_delete(outdir)

# Run interspecies DIAMOND search
outdir2 <- file.path(tempdir(), "benchmark3_inter")
compare_df <- data.frame(
    query = "arabidopsis.thaliana", target = "amborella.trichopoda"
)

diamond_inter <- syntenet::run_diamond(
    seq = pdata$seq,
    compare = compare_df,
```

```
    outdir = outdir2,
    threads = 4,
    ... = "--sensitive"
)
fs::dir_delete(outdir2)
```

Now, let's classify duplicates with **doubletrouble**.

```
# Classify duplicates with doubletrouble
start1 <- Sys.time()
dups1 <- classify_gene_pairs(
    blast_list = diamond_intra[2],
    annotation = pdata$annotation,
    blast_inter = diamond_inter,
    scheme = "extended",
    collinearity_dir = here("products")
)
end1 <- Sys.time()
runtime1 <- end1 - start1
```

Next, we will classify duplicates with DupGen_finder. For that, we will first export input data in the required format.

```
# Export data
## .blast files
b1 <- diamond_intra$arabidopsis.thaliana_arabidopsis.thaliana |>
    mutate(
        query = str_replace_all(query, "^ara_", ""),
        db = str_replace_all(db, "^ara_", "")
    )

b2 <- diamond_inter$arabidopsis.thaliana_amborella.trichopoda |>
    mutate(
        query = str_replace_all(query, "^ara_", ""),
        db = str_replace_all(db, "^amb_", "")
    )

write_tsv(b1, file = file.path(tempdir(), "Ath.blast"), col_names = FALSE)
write_tsv(b2, file = file.path(tempdir(), "Ath_Atr.blast"), col_names = FALSE)

## .gff files
gff1 <- pdata$annotation$arabidopsis.thaliana |>
    as.data.frame() |>
    mutate(gene = str_replace_all(gene, "^ara_", "")) |>
    mutate(seqnames = str_replace_all(seqnames, "ara_", "ara-")) |>
    dplyr::select(seqnames, gene, start, end)

gff2 <- pdata$annotation$amborella.trichopoda |>
    as.data.frame() |>
    mutate(gene = str_replace_all(gene, "^amb_", "")) |>
    mutate(seqnames = str_replace_all(seqnames, "^amb_", "amb-")) |>
    dplyr::select(seqnames, gene, start, end)
```

```r
gff2 <- bind_rows(gff1, gff2)

write_tsv(gff1, file = file.path(tempdir(), "Ath.gff"), col_names = FALSE)
write_tsv(gff2, file = file.path(tempdir(), "Ath_Atr.gff"), col_names = FALSE)

# Classify duplicates with DupGen_finder
args = c(
    "-i", tempdir(), "-t Ath -c Atr",
    "-o", file.path(tempdir(), "results"),
    "-e 1e-10"
)

start2 <- Sys.time()
system2("DupGen_finder.pl", args = args)
end2 <- Sys.time()
runtime2 <- end2 - start2
```

Now, let's compare both algorithms in terms of runtime and results.

```r
# Load `DupGen_finder.pl` results
files <- c(
    "Ath.wgd.pairs", "Ath.tandem.pairs", "Ath.proximal.pairs",
    "Ath.transposed.pairs", "Ath.dispersed.pairs"
)
files <- file.path(tempdir(), "results", files)
names(files) <- c("SD", "TD", "PD", "TRD", "DD")
dups2 <- Reduce(rbind, lapply(seq_along(files), function(x) {
    d <- read_tsv(files[x], show_col_types = FALSE) |>
        mutate(type = names(files)[x]) |>
        select(1, 3, type) |>
        as.data.frame()

    names(d)[c(1,2)] <- c("dup1", "dup2")

    return(d)
}))

# Compare runtime
comp_runtime <- data.frame(doubletrouble = runtime1, DupGen_finder = runtime2)

# Compare number of gene pairs per category
comp_results <- inner_join(
    count(dups1$arabidopsis.thaliana, type) |>
        dplyr::rename(n_doubletrouble = n),
    count(dups2, type) |>
        dplyr::rename(n_DupGen_finder = n),
    by = "type"
)
comp_results <- bind_rows(
    comp_results,
    data.frame(
```

```
            type = "Total",
            n_doubletrouble = sum(comp_results$n_doubletrouble),
            n_DupGen_finder = sum(comp_results$n_DupGen_finder)
        )
)
```

```
list(runtime = comp_runtime, results = comp_results)
## $runtime
##   doubletrouble DupGen_finder
## 1  3.71474 secs 3.373682 secs
##
## $results
##    type n_doubletrouble n_DupGen_finder
## 1    SD            4329            4355
## 2    TD            2075            2131
## 3    PD            2789             896
## 4   TRD            6703            5941
## 5    DD           31589           17834
## 6 Total           47485           31157
```

Overall, results are similar, but there are important differences. In terms of runtime, there is no significant difference (this is the runtime of a single run, so there's some stochasticity). In terms of results, the numbers of SD, TD, and TRD pairs are similar, but there are more pronounced differences for PD and DD pairs. In particular, the total number of paralogous pairs differs between algorithms. When we remove rows from the DIAMOND output based on the E-value threshold and remove self (e.g., "gene1-gene1") and redundant hits (e.g., "gene1-gene2" and "gene2-gene1"), we get the following total number of pairs:

```
# Get total number of paralog pairs from DIAMOND output
evalue <- 1e-10
dmd <- diamond_intra$arabidopsis.thaliana_arabidopsis.thaliana

all_paralogs <- lapply(list(dmd), function(x) {
    fpair <- x[x$evalue <= evalue, c(1, 2)]
    fpair <- fpair[fpair[, 1] != fpair[, 2], ]
    fpair <- fpair[!duplicated(t(apply(fpair, 1, sort))), ]
    names(fpair) <- c("dup1", "dup2")
    return(fpair)
})[[1]]
```

```
nrow(all_paralogs)
## [1] 47485
```

The total number of paralogous pairs in the DIAMOND output is the same as sum of classes for **doubletrouble**, but greater than the sum of classes for **DupGen_finder**, indicating that the latter probably does some additional (undocumented) filtering before classifying gene pairs, or it could be a bug.

Finally, since the number of PD pairs identified by **doubletrouble** is much greater than the number of PD pairs identified by **DupGen_finder**, we will explore a few of these PD pairs so check whether **doubletrouble** misclassified them.

```
pd1 <- dups1$arabidopsis.thaliana |>
    filter(type == "PD")

pd2 <- dups2 |>
    filter(type == "PD")

# Get all pairs in `pd1` and `pd2`
p1 <- t(apply(pd1[, 1:2], 1, sort)) |>
    as.data.frame() |>
    mutate(V1 = str_replace_all(V1, "^ara_", "")) |>
    mutate(V2 = str_replace_all(V2, "^ara_", "")) |>
    mutate(pair_string = str_c(V1, V2, sep = "-")) |>
    pull(pair_string)

p2 <- t(apply(pd2[, 1:2], 1, sort)) |>
    as.data.frame() |>
    mutate(pair_string = str_c(V1, V2, sep = "-")) |>
    pull(pair_string)

# Sample PD pairs from doubletrouble that are not PD pairs in DupGen_finder
examples <- p1[!p1 %in% p2] |> head(n = 5)

# Check whether they are PD pairs or not
ath_annot <- pdata$annotation$arabidopsis.thaliana
range_examples <- lapply(examples, function(x) {
    g1 <- paste0("ara_", strsplit(x, "-")[[1]][1])
    g2 <- paste0("ara_", strsplit(x, "-")[[1]][2])
    ranges <- ath_annot[ath_annot$gene %in% c(g1, g2)]
    return(ranges)
})
```

```
range_examples
## [[1]]
## GRanges object with 2 ranges and 1 metadata column:
##       seqnames        ranges strand |          gene
##          <Rle>     <IRanges>  <Rle> |   <character>
##   128    ara_1 428650-430720      - | ara_AT1G02220
##   130    ara_1 437860-439559      - | ara_AT1G02250
##   -------
##   seqinfo: 7 sequences from an unspecified genome; no seqlengths
##
## [[2]]
## GRanges object with 2 ranges and 1 metadata column:
##       seqnames        ranges strand |          gene
##          <Rle>     <IRanges>  <Rle> |   <character>
##   127    ara_1 427548-427811      - | ara_AT1G02210
##   130    ara_1 437860-439559      - | ara_AT1G02250
##   -------
##   seqinfo: 7 sequences from an unspecified genome; no seqlengths
##
## [[3]]
```

```
## GRanges object with 2 ranges and 1 metadata column:
##         seqnames        ranges strand |          gene
##            <Rle>     <IRanges>  <Rle> |   <character>
##   17117    ara_4 656407-659178      - | ara_AT4G01520
##   17120    ara_4 673862-676445      - | ara_AT4G01550
##   -------
##   seqinfo: 7 sequences from an unspecified genome; no seqlengths
##
## [[4]]
## GRanges object with 2 ranges and 1 metadata column:
##         seqnames            ranges strand |          gene
##            <Rle>         <IRanges>  <Rle> |   <character>
##   15329    ara_3 17882465-17884515      + | ara_AT3G48290
##   15332    ara_3 17887996-17889942      + | ara_AT3G48310
##   -------
##   seqinfo: 7 sequences from an unspecified genome; no seqlengths
##
## [[5]]
## GRanges object with 2 ranges and 1 metadata column:
##         seqnames            ranges strand |          gene
##            <Rle>         <IRanges>  <Rle> |   <character>
##   25845    ara_5 21378702-21379744      + | ara_AT5G52720
##   25847    ara_5 21382482-21383432      + | ara_AT5G52740
##   -------
##   seqinfo: 7 sequences from an unspecified genome; no seqlengths
```

In these first five examples of pairs that are classified as PD pairs by **doubletrouble**, but not by **DupGen_finder**, we can see based on the numbers in row names that they are indeed very close, separated by only a few genes (<10). Thus, they are true PD pairs that **DupGen_finder** failed to classify as PD pairs, or removed in their undocumented filtering (described above).

Session info

This document was created under the following conditions:

```
## - Session info ---------------------------------------------------------------
##  setting  value
##  version  R version 4.4.1 (2024-06-14)
##  os       Ubuntu 22.04.4 LTS
##  system   x86_64, linux-gnu
##  ui       X11
##  language (EN)
##  collate  en_US.UTF-8
##  ctype    en_US.UTF-8
##  tz       Europe/Brussels
##  date     2024-07-26
##  pandoc   3.1.1 @ /usr/lib/rstudio/resources/app/bin/quarto/bin/tools/ (via rmarkdown)
##
## - Packages -------------------------------------------------------------------
```

Supplementary Text S5: Runtime benchmark

```
##  package              * version   date (UTC) lib source
##  abind                  1.4-5     2016-07-21 [1] CRAN (R 4.4.1)
##  ade4                   1.7-22    2023-02-06 [1] CRAN (R 4.4.1)
##  AnnotationDbi          1.66.0    2024-05-01 [1] Bioconductor 3.19 (R 4.4.1)
##  ape                    5.8       2024-04-11 [1] CRAN (R 4.4.1)
##  Biobase                2.64.0    2024-04-30 [1] Bioconductor 3.19 (R 4.4.1)
##  BiocGenerics           0.50.0    2024-04-30 [1] Bioconductor 3.19 (R 4.4.1)
##  BiocIO                 1.14.0    2024-04-30 [1] Bioconductor 3.19 (R 4.4.1)
##  BiocManager            1.30.23   2024-05-04 [1] CRAN (R 4.4.1)
##  BiocParallel           1.38.0    2024-04-30 [1] Bioconductor 3.19 (R 4.4.1)
##  BiocStyle            * 2.32.1    2024-06-16 [1] Bioconductor 3.19 (R 4.4.1)
##  Biostrings             2.72.1    2024-06-02 [1] Bioconductor 3.19 (R 4.4.1)
##  bit                    4.0.5     2022-11-15 [1] CRAN (R 4.4.1)
##  bit64                  4.0.5     2020-08-30 [1] CRAN (R 4.4.1)
##  bitops                 1.0-7     2021-04-24 [1] CRAN (R 4.4.1)
##  blob                   1.2.4     2023-03-17 [1] CRAN (R 4.4.1)
##  bookdown               0.40      2024-07-02 [1] CRAN (R 4.4.1)
##  cachem                 1.1.0     2024-05-16 [1] CRAN (R 4.4.1)
##  cli                    3.6.3     2024-06-21 [1] CRAN (R 4.4.1)
##  coda                   0.19-4.1  2024-01-31 [1] CRAN (R 4.4.1)
##  codetools              0.2-20    2024-03-31 [1] CRAN (R 4.4.1)
##  colorspace             2.1-0     2023-01-23 [1] CRAN (R 4.4.1)
##  crayon                 1.5.3     2024-06-20 [1] CRAN (R 4.4.1)
##  curl                   5.2.1     2024-03-01 [1] CRAN (R 4.4.1)
##  DBI                    1.2.3     2024-06-02 [1] CRAN (R 4.4.1)
##  DelayedArray           0.30.1    2024-05-07 [1] Bioconductor 3.19 (R 4.4.1)
##  digest                 0.6.36    2024-06-23 [1] CRAN (R 4.4.1)
##  doParallel             1.0.17    2022-02-07 [1] CRAN (R 4.4.1)
##  doubletrouble        * 1.4.1     2024-07-25 [1] Bioconductor
##  dplyr                * 1.1.4     2023-11-17 [1] CRAN (R 4.4.1)
##  evaluate               0.24.0    2024-06-10 [1] CRAN (R 4.4.1)
##  fansi                  1.0.6     2023-12-08 [1] CRAN (R 4.4.1)
##  fastmap                1.2.0     2024-05-15 [1] CRAN (R 4.4.1)
##  forcats              * 1.0.0     2023-01-29 [1] CRAN (R 4.4.1)
##  foreach                1.5.2     2022-02-02 [1] CRAN (R 4.4.1)
##  generics               0.1.3     2022-07-05 [1] CRAN (R 4.4.1)
##  GenomeInfoDb           1.40.1    2024-05-24 [1] Bioconductor 3.19 (R 4.4.1)
##  GenomeInfoDbData       1.2.12    2024-07-24 [1] Bioconductor
##  GenomicAlignments      1.40.0    2024-04-30 [1] Bioconductor 3.19 (R 4.4.1)
##  GenomicFeatures        1.56.0    2024-04-30 [1] Bioconductor 3.19 (R 4.4.1)
##  GenomicRanges          1.56.1    2024-06-12 [1] Bioconductor 3.19 (R 4.4.1)
##  ggnetwork              0.5.13    2024-02-14 [1] CRAN (R 4.4.1)
##  ggplot2              * 3.5.1     2024-04-23 [1] CRAN (R 4.4.1)
##  glue                   1.7.0     2024-01-09 [1] CRAN (R 4.4.1)
##  gtable                 0.3.5     2024-04-22 [1] CRAN (R 4.4.1)
##  here                 * 1.0.1     2020-12-13 [1] CRAN (R 4.4.1)
##  hms                    1.1.3     2023-03-21 [1] CRAN (R 4.4.1)
##  htmltools              0.5.8.1   2024-04-04 [1] CRAN (R 4.4.1)
##  htmlwidgets            1.6.4     2023-12-06 [1] CRAN (R 4.4.1)
##  httr                   1.4.7     2023-08-15 [1] CRAN (R 4.4.1)
##  igraph                 2.0.3     2024-03-13 [1] CRAN (R 4.4.1)
```

```
##  intergraph            2.0-4     2024-02-01 [1] CRAN (R 4.4.1)
##  IRanges               2.38.1    2024-07-03 [1] Bioconductor 3.19 (R 4.4.1)
##  iterators             1.0.14    2022-02-05 [1] CRAN (R 4.4.1)
##  jsonlite              1.8.8     2023-12-04 [1] CRAN (R 4.4.1)
##  KEGGREST              1.44.1    2024-06-19 [1] Bioconductor 3.19 (R 4.4.1)
##  knitr                 1.48      2024-07-07 [1] CRAN (R 4.4.1)
##  lattice               0.22-6    2024-03-20 [1] CRAN (R 4.4.1)
##  lifecycle             1.0.4     2023-11-07 [1] CRAN (R 4.4.1)
##  lubridate           * 1.9.3     2023-09-27 [1] CRAN (R 4.4.1)
##  magrittr              2.0.3     2022-03-30 [1] CRAN (R 4.4.1)
##  MASS                  7.3-61    2024-06-13 [1] CRAN (R 4.4.1)
##  Matrix                1.7-0     2024-04-26 [1] CRAN (R 4.4.1)
##  MatrixGenerics        1.16.0    2024-04-30 [1] Bioconductor 3.19 (R 4.4.1)
##  matrixStats           1.3.0     2024-04-11 [1] CRAN (R 4.4.1)
##  mclust                6.1.1     2024-04-29 [1] CRAN (R 4.4.1)
##  memoise               2.0.1     2021-11-26 [1] CRAN (R 4.4.1)
##  MSA2dist              1.8.0     2024-04-30 [1] Bioconductor 3.19 (R 4.4.1)
##  munsell               0.5.1     2024-04-01 [1] CRAN (R 4.4.1)
##  network               1.18.2    2023-12-05 [1] CRAN (R 4.4.1)
##  networkD3             0.4       2017-03-18 [1] CRAN (R 4.4.1)
##  nlme                  3.1-165   2024-06-06 [1] CRAN (R 4.4.1)
##  patchwork           * 1.2.0     2024-01-08 [1] CRAN (R 4.4.1)
##  pheatmap              1.0.12    2019-01-04 [1] CRAN (R 4.4.1)
##  pillar                1.9.0     2023-03-22 [1] CRAN (R 4.4.1)
##  pkgconfig             2.0.3     2019-09-22 [1] CRAN (R 4.4.1)
##  png                   0.1-8     2022-11-29 [1] CRAN (R 4.4.1)
##  purrr               * 1.0.2     2023-08-10 [1] CRAN (R 4.4.1)
##  pwalign               1.0.0     2024-04-30 [1] Bioconductor 3.19 (R 4.4.1)
##  R6                    2.5.1     2021-08-19 [1] CRAN (R 4.4.1)
##  RColorBrewer          1.1-3     2022-04-03 [1] CRAN (R 4.4.1)
##  Rcpp                  1.0.13    2024-07-17 [1] CRAN (R 4.4.1)
##  RCurl                 1.98-1.16 2024-07-11 [1] CRAN (R 4.4.1)
##  readr               * 2.1.5     2024-01-10 [1] CRAN (R 4.4.1)
##  restfulr              0.0.15    2022-06-16 [1] CRAN (R 4.4.1)
##  rjson                 0.2.21    2022-01-09 [1] CRAN (R 4.4.1)
##  rlang                 1.1.4     2024-06-04 [1] CRAN (R 4.4.1)
##  rmarkdown             2.27      2024-05-17 [1] CRAN (R 4.4.1)
##  rprojroot             2.0.4     2023-11-05 [1] CRAN (R 4.4.1)
##  Rsamtools             2.20.0    2024-04-30 [1] Bioconductor 3.19 (R 4.4.1)
##  RSQLite               2.3.7     2024-05-27 [1] CRAN (R 4.4.1)
##  rstudioapi            0.16.0    2024-03-24 [1] CRAN (R 4.4.1)
##  rtracklayer           1.64.0    2024-04-30 [1] Bioconductor 3.19 (R 4.4.1)
##  S4Arrays              1.4.1     2024-05-20 [1] Bioconductor 3.19 (R 4.4.1)
##  S4Vectors             0.42.1    2024-07-03 [1] Bioconductor 3.19 (R 4.4.1)
##  scales                1.3.0     2023-11-28 [1] CRAN (R 4.4.1)
##  seqinr                4.2-36    2023-12-08 [1] CRAN (R 4.4.1)
##  sessioninfo           1.2.2     2021-12-06 [1] CRAN (R 4.4.1)
##  SparseArray           1.4.8     2024-05-24 [1] Bioconductor 3.19 (R 4.4.1)
##  statnet.common        4.9.0     2023-05-24 [1] CRAN (R 4.4.1)
##  stringi               1.8.4     2024-05-06 [1] CRAN (R 4.4.1)
##  stringr             * 1.5.1     2023-11-14 [1] CRAN (R 4.4.1)
```

```
##  SummarizedExperiment   1.34.0    2024-05-01 [1] Bioconductor 3.19 (R 4.4.1)
##  syntenet               1.6.0     2024-05-01 [1] Bioconductor 3.19 (R 4.4.1)
##  tibble               * 3.2.1     2023-03-20 [1] CRAN (R 4.4.1)
##  tidyr                * 1.3.1     2024-01-24 [1] CRAN (R 4.4.1)
##  tidyselect             1.2.1     2024-03-11 [1] CRAN (R 4.4.1)
##  tidyverse            * 2.0.0     2023-02-22 [1] CRAN (R 4.4.1)
##  timechange             0.3.0     2024-01-18 [1] CRAN (R 4.4.1)
##  tzdb                   0.4.0     2023-05-12 [1] CRAN (R 4.4.1)
##  UCSC.utils             1.0.0     2024-04-30 [1] Bioconductor 3.19 (R 4.4.1)
##  utf8                   1.2.4     2023-10-22 [1] CRAN (R 4.4.1)
##  vctrs                  0.6.5     2023-12-01 [1] CRAN (R 4.4.1)
##  withr                  3.0.0     2024-01-16 [1] CRAN (R 4.4.1)
##  xfun                   0.46      2024-07-18 [1] CRAN (R 4.4.1)
##  XML                    3.99-0.17 2024-06-25 [1] CRAN (R 4.4.1)
##  XVector                0.44.0    2024-04-30 [1] Bioconductor 3.19 (R 4.4.1)
##  yaml                   2.3.9     2024-07-05 [1] CRAN (R 4.4.1)
##  zlibbioc               1.50.0    2024-04-30 [1] Bioconductor 3.19 (R 4.4.1)
##
##  [1] /home/faalm/R/x86_64-pc-linux-gnu-library/4.4
##  [2] /usr/local/lib/R/site-library
##  [3] /usr/lib/R/site-library
##  [4] /usr/lib/R/library
##
## ------------------------------------------------------------------------------
```
